# Causally Mapping the Cerebellum in Children and Young Adults: from Motor to Cognition

**DOI:** 10.1007/s12311-024-01778-8

**Published:** 2025-01-16

**Authors:** Marina Veyrie, Bertrand Beffara, Nathalie Richard, Carmine Mottolese, Alexandru Szathmari, Federico Di Rocco, Cécile Faure Conter, Pierre Leblond, Michel Desmurget, Irene Cristofori, Pierre-Aurélien Beuriat

**Affiliations:** 1 Institute of Cognitive Science Marc Jeannerod, CNRS/UMR 5229, 69500 Bron, France; 2 Université, Claude Bernard Lyon 1, 69100 Villeurbanne, France; 3 Department of Pediatric Neurosurgery, Hôpital Femme Mère Enfant, 69500 Bron, France; 4 Institut d’Hématologie Et d’Oncologie Pédiatrique, Lyon, France; 5 Institut National de La Santé Et de La Recherche Médicale, Lyon, France

**Keywords:** Lesion mapping, Cerebellum, Motricity, Cognition

## Abstract

While the cerebellum’s role in orchestrating motor execution and routines is well established, its functional role in supporting cognition is less clear. Previous studies claim that motricity and cognition are mapped in different areas of the cerebellar cortex, with an anterior/posterior dichotomy. However, most of the studies supporting this claim either use correlational methods (neuroimaging) or are lesion studies that did not consider central covariates (such as age, gender, treatment presence, and deep nuclei impairment) known to influence motor and cognitive recoveries in patients. Here, we used voxel-based lesion-symptom mapping (VLSM) on children and young adults having undergone cerebellar tumor resection. This approach allows to control for these covariates and evaluate causal relationships between brain anatomy and behavioral performances to disentangle the anatomic substrate of motor and cognitive functions. VLSM analyses showed that both motor and cognitive impairments were greater in children and young adults with lesions of the posterior cerebellum. These results highlight distinct and overlapping structural correlates of motor and cognitive performance in the cerebellum and are consistent with structural and functional hypotheses of integration of the cerebellum in motor and cognitive functions.

**Supplementary Information:**

The online version contains supplementary material available at 10.1007/s12311-024-01778-8.

## Introduction

Historically, the cerebellum has been considered as a key structure for motor control. Its role in orchestrating the timing and coordination of motor outputs and motor learning [22, 23], in interaction with different cortical structures [4, 37, 40], is well-established. In particular, lesion studies have shown that damages to the cerebellum can lead to ataxia (i.e. uncoordinated movements, see [1]), but also to more generalized deficits such as walking or keeping balance [36]. Neuroimaging studies identified relationships between the dysfunction of specific cerebellar regions and the onset of motor difficulties (e.g. anterior lobe and lobules VIII and IX, see [33],anterior lobe and adjacent lobule VI, see [51]). Functional correlates of motor control during specific tasks were also described (e.g. lobe VIIIa and b during finger tapping, see [54],central lobules II-III for the foot task and hemispheric lobules IV-V for the hand task, see [39]), as well as the existence of efferent representation associated with different body parts in different lobules (e.g. face/mouth motor mapping in lobule VI, see [38]).

Besides the well-established involvement of the cerebellum in motor control, there is now accumulating evidence that this structure also plays a crucial role in multiple cognitive [7, 20, 45] and social cognition [8, 9, 61–64]. This conclusion is supported by anatomical mappings of the cerebellum’s outputs to non-motor cortical regions involved in cognitive processing [15, 18, 56] and functional connectivity studies [14, 67].

During the last two decades, influential articles have advocated the existence of an anatomo-functional dichotomy between the anterior and the posterior cerebellum, with a privileged role of the anterior cerebellum for motricity and the posterior cerebellum for cognition [45, 52–54]. This model is supported by evolutionary differences between the anterior and posterior cerebellum [3] and by similar expansions of the posterior cerebellum and associative areas through evolution [45]. However, this dichotomy is challenged by studies showing that motor functions were also associated with the posterior parts of the cerebellar cortex on the one hand [38, 44, 48] and, more importantly, that regions of the anterior cerebellum could be involved in higher cognitive functions such as mentalizing [65], see also [62, 63], cognitive impairments in schizophrenic patients [28] and executive functions [25]. A recent and extensive fMRI study [32] further supported this view. The main strengths of their study are the large sample size (186 participants), the diversity of the cognitive tasks employed (26 different tasks) and the fact that they separately modeled the effect of motor-related vs. cognitive-related hemodynamic activity, thus allowing for a robust assessment of the involvement of the cerebellum in cognition. This emerging evidence questions the clear anterior–posterior aforementioned cerebellar functional dichotomy. While it is well admitted that the posterior cerebellum also contains sensorimotor representations [38, 44, 45, 48], the involvement of the anterior cerebellum in cognition is less accepted [45]. As a consequence, the idea of the anterior cerebellum as “the sensorimotor cerebellum” and of the posterior as the “cognitive/limbic” cerebellum [45] still permeates (see also [47, 53]).

The present study tackles this issue using a causal method, the Voxel-based lesion-symptom mapping (VLSM; [6]). This approach constitutes a powerful methodology to address anatomo-functional relationships in patients suffering from brain lesions. It does not consider predefined regions of interest but identifies all brain regions whose lesions are correlated with functional deficits of interest [6]. To date, VLSM has been widely used in the context of surgical resection of cortical tumors [42]. However, only a few studies [25], see also [48] who recruited cerebellar tumor and stroke patients) have used it in the context of cerebellar tumors. For instance, Grosse et al. [25] used VLSM to assess the relationship between cerebellum lesions and motor/cognitive tasks. They found that lesions in both the anterior and posterior cerebellum were associated with both motor and cognitive deficits in pediatric cerebellar brain tumor survivors.

A possible explanation for the contradictory observations provided concerning the existence of a motor/cognitive anatomo-functional dichotomy within the cerebellum, could be related to the existence of uncontrolled covariates by previous studies. For example, the patient’s age at the time of surgical operation can be critical for recovery: Beuriat, Cristofori, et al. (2020) showed that a surgical operation before 7 years old can induce delays in the acquisition and recovery of motor and cognitive functions [10]. In addition, it is now well-acknowledged that radiotherapy can affect neuropsychological outcomes ("radiation-induced cognitive impairment", see e.g. [5, 24, 35]). Another crucial aspect is the selection of neuropsychological tests requiring good scientific validity. For instance, for motricity, the Purdue Pegboard (PPT, [57]) has content validity, proving that it correctly measures its target. On the other hand, it has been shown that this test involves different degrees of motor execution complexity from the simple one-hand to the assembly tests, proving that the Purdue Pegboard measures distinct factors, motor coordination, execution speed, and motor planning, acquired at distinct times during development (Beguet & Albaret., 1998).

Based on these limitations, the objective of the present study is to use VLSM while controlling for the main covariates that are known to influence clinical recovery after cerebellar lesion including 1) age at surgery (Beuriat, Cristofori, et al., 2020), 2) gender (i.e. the norms of the neuropsychological tests used in the current study depend on gender), 3) presence of lesions of the deep cerebellar nuclei [48] who were the first to report that damages to cerebellar nuclei lead to long-term motor deficits) – that has been identified as risk factor for pediatric cerebellar cognitive and affective syndrome [2] –, and 4) administration of postsurgical treatment (such as radiotherapy or chemotherapy) (Beuriat, Cristofori, et al., 2020). Also, we used several complementary fine-grained evaluations of motor and cognitive functions (Purdue Pegboard, ataxia questionnaire, and intelligence scale and subscales). Applying all these rigorous controls, we expect to clarify the ongoing controversy of whether there is an anterior/posterior anatomical dissociation between motor and cognitive processing, within the cerebellum. We hypothesize that a clear cognitive vs. motor deficit distinction is not possible only based on the anterior vs. posterior cerebellum lesion location [32].

## Materials and Methods

### Patients

For this study, 40 patients (18 females), aged from 1 to 19 years old, operated under general anesthesia between 2001 and 2016 (aged at surgery 9,46 ± 4,68 (min = 1, max = 19)) at the Women-Mother–Child Hospital (Lyon, France) were recruited (see Table 1 for the patients’ characteristics).

**Table 1:** Patients demographic characteristics

N	*40 (18F); (22M)*
Age at surgery for all participants (years) *(mean* ± *SD)*	9,46 ± 4.68 (min: 1 max: 19)
Toddlers (< 6 years) (N)	11
School-aged children (6–12) (N)	18
Adolescents (13–20) (N)	11
Age at neuropsychological assessment (years) *(mean* ± *SD)*	14.40 ± 5.09 (min: 5 max: 23)
Hand laterality before surgery	37 (right); 3 (left)
Hand laterality after surgery	31 (right); 9 (left)
Lesion volume *(mean* ± *SD)*	41,970 mm^3^ ± 30,750
Hydrocephalus at diagnosis (N)	19
Treatment after surgery (radio/chemotherapy) (N)	20
Deep nuclei impairment (N)	15
Type of the tumors	14 (astrocytomas); 4 (epandymomas); 2 (gangliocytomas); 3 (hemangioblastomas); 16 (medulloblastomas) and 1 (arteriovenous malformation)

Patients were invited to participate in a long-term follow-up study, in addition to their standard clinical evaluation. The study was performed under the patients’ (or their parents for participant under legal age) formal consent, with the approbation of the local institutional ethical committee and with the precepts of the Declaration of Helsinki. Neuropsychological tests were administered between 3 to 5 years after surgery.

Based on Beuriat, Cristofori et al. (2020), we included the following inclusion criteria: patients (i) underwent the excision of a cerebellar tumor before testing (from 3 to 5 years before) (ii) had a total tumor removal and did not present any recurrence; (iii) were not undergoing medical treatments at the time of the evaluation; (iv) were not suffering from transient postoperative complications likely to interfere with recovery, such as cerebellar mutism (to avoid clinical disparities between patients) and (v) used French as their mother tongue.

Evaluations were completed under the supervision of a qualified neuropsychologist (MD) and clinician (PAB) who were blind to the patients’ clinical history and imaging results.

Treatment after surgery (yes or no), deep nuclei impairment (yes or no), gender and age at surgery were entered as covariates. We used the model comprising all these covariates as the main analysis in the manuscript, and included the results of alternative models as supplementary tables (Supplementary Tables 1–2).

### Motor and Cognitive Assessments

To assess fine motor functions, the Purdue Pegboard test (PPT, [57]), was used. To assess ataxia deficits, the International cooperative ataxia rating scale (ICARS, [58]) was used. To assess cognition, the general intelligence scales were used, different versions depending on patients’ age (Wechsler intelligence scale (WAIS-IV and WISC-V French versions, see [68, 69]). In the following sections, we describe in more detail the tests/scales we used.

### Motricity—Purdue Pegboard Test

The PPT is a manual exercise that consists of placing small rods, washers, and nuts on a pegboard as quickly as possible. The first step consists of using only the dominant hand, the second step only the non-dominant hand, and the third step both hands simultaneously. The fourth step involves the assembly of a structure composed of different parts (rods, washers, and nuts), using both hands simultaneously. This last exercise involves a cognitive dimension (working memory, programming). The score is the number of pieces placed on the board in 30 s for the first 3 stages and 60 s for the fourth (a higher score represents better fine motor ability). The final score is obtained by averaging three consecutive repetitions for each step and then a Z-score is calculated by using the Purdue Pegboard scoring app provided by the Lafayette Instrument Company (www.lafayetteinstrument.com).

### Evaluation of Ataxia – ICARS

The ICARS is used to quantify the level of impairment from zero (no ataxia) to 100 (severe ataxia). It is a 100-points semi-quantitative scale evaluating different components of cerebellar symptoms, such as postural and gait disturbances, limb ataxia, dysarthria and oculomotor disorders. To assess deficits in these different areas, 19 items are proposed, such as walking capacity, gait speed, and standing capacity. For each item, a score represents the patient’s difficulty in the given motor skill. The sum of the scores for each 19 items is then calculated and leads a total score and different subscores, such as the kinetic function, posture and gait, oculomotor disorders, speech. Patients with no motor impairments have a total score below 7 [55]. We used the raw score for each subtest for the VLSM analyses.

### Cognition – Wechsler Iintelligence Scales

For the evaluation of the intelligence quotient, the Wechsler intelligence scale was used (WAIS-IV for patients older than 16 years old and WISC-V for patients younger than 16 years old). All mandatory subtests were administrated. Following this, we were able to obtain scores for the following indices: Full scale IQ (FSIQ) (based on the total combined performances of the following indices: (1) Perceptual reasoning (PRI) reflects reasoning skills and ability to interpret, organize and understand visual information, (2) Working memory (WMI), the ability to take in and hold information, and to perform a mental operation on this information (3) Processing speed (PSI) measures visual and motor speed (found visual information quickly and efficiently), (4) Verbal comprehension (VCI), assess verbal skills for understanding, use and think spoken language. Full scale score and subscale scores were used as outcomes in the VLSM analyses.

## Imaging

For each patient, MRI were performed during the clinical visit at the hospital, before surgery (approximatively 5 years before neuropsychological assessment). Imaging consisted in T1 weighted scans with and without gadolinium used to delimit tumor volume. Acquisitions were performed using a 1.5 Tesla magnetic resonance scanner (Philips, NV) using 3D acquisition with 1 mm slices.

The MRI used for this study were the clinical MRI performed for the standard of care. Therefore, the quality was control by the radiologist the day of the scan. For the youngest patients or the ones who could not perform the MRI because of hustle, a general anesthesia was performed to ensure a good image quality without movement artifacts.

Pre-operative MRI images were used to delineate the lesion extent (due to the cerebellum’s tendency to collapse after tumor resection, it is challenging to trace lesions in post-operative images).

### Voxel‑based Lesion Symptom Mapping

Anatomical normalization of the cerebellum was performed on preoperative MRI images with the SPM12b toolbox of the matlab software (https:// www.fil.ion.ucl.ac.uk/ spm/) for preprocessing, and standardize the MRI data in the MNI (Montreal neurological Institute) space, as well as to reset the origin if needed, and realign the images.

All cerebellar lesions were drawn manually by PAB (associate professor and pediatric neurosurgeon) using the MRIcron software in order to obtain the volume of interest (VOI) on the T1-weighted MRI scans.

VOI, clinical and behavioral data were then analyzed using a voxel‑based lesion symptom mapping (VLSM) procedure. The analyses were carried out using the VLSM package version 2.60 (https://aphasialab.org/vlsm/) on MATLAB R2017a (Mathworks, Natick, MA) software. Identification of the brain regions associated with the significant voxels was made using the MNI2atlas on Matlab (https://fr.mathworks.com/matlabcentral/fileexchange/87047-mni2atlas). Deep cerebellar nuclei were identified using the Diedrichsen atlas (https://www.diedrichsenlab.org/imaging/propatlas.htm; [17]. Outcomes of these automatic procedures were visually reviewed on individual MRI by the neurosurgeons of the team (CM, AS, FDR, and PAB).

The behavioral outcomes in the VLSM analysis were the Purdue Pegboard test scores, the ICARS score, and the Full intelligence score and subscores [Perceptual reasoning (PRI) Working memory (WMI), Processing speed (PSI) and Verbal comprehension (VCI)]. In addition, patient age, gender, treatment (radiotherapy or chemotherapy), and deep nuclei impairment (the type of radiation was not considered, as most children who received radiation underwent standard radiation rather than proton therapy) were used as covariables/factors, to account for the possible influence of those variables.

Once the lesions had been traced and the motor and cognitive data obtained neuropsychological assessments, we carried out the VLSM analyses, we used the Matlab's VLSM2 toolbox (https://aphasialab.org/vlsm/), using a file containing all images with lesion tracing, and a file containing all neuropsychological test scores for each patient as inputs. VLSM analyses were only conducted when at least ten patients had a lesion for a given voxel (e.g. see [21]). The alpha significance level for the voxel-by-voxel comparisons was set to 0.01 based on previous VLSM studies (Dal [16, 21, 31, 59]). For each voxel, the toolbox compares neuropsychological data for patients having a lesion there vs. not having a lesion at this location (t-tests performed independently for each voxel). Correction for multiple comparisons was achieved by permutation analyses. Statistical maps were generated for 1000 random assignments of behavioural scores to patients, with the maximum cluster size recorded each time. We used the fifth percentile maximum cluster size from the 1000 permutations as the minimum cluster size, thus ensuring corrected cluster size significance of *p* < 0.05.

The behavioral outcomes in the VLSM analysis were the Purdue Pegboard test (z-score), ICARS (raw score, a higher score implies worse performances), and Wechsler intelligence scores (standard score, a higher score implies better performances). To account for possible confounds, we used age at surgery, gender, presence of treatment after the surgery (radiotherapy or chemotherapy) and impairment of deep nuclei as covariates. In our study, 15 of the 40 patients have a lesion of the deep nuclei of the cerebellum.

## Results

### Neuropsychological Assessment

At the Purdue Pegboard test, 16 patients had an impaired z-score (i.e., above 2 SD) for the Purdue Pegboard right-hand subtest.10 patients had an impaired z-score for the left-hand subtest. 17 for the both hands subtest and 14 for the assembly subtest.

For the ICARS, 19 patients had an impaired raw-score (lower than 7 as suggested by [55]) for the total score subtests, 8 for kinetic function, 4 for posture and gait, 0 for oculomotor disorders and 0 for speech.

For the Wechsler intelligence scale (Wechsler 2008; 2014), raw scores were converted to standard scores using age and gender reference norms (Wechsler 2008; 2014). 4 patients had a score below the norm for the full score intelligence (FSIQ), 1 for fluid reasoning (FRI), 3 for working memory (WMI), 5 for processing speed (PSI) and 2 for verbal comprehension (VCI) (see Table 2 for details regarding the scores to the neuropsychological assessments).

**Table 2:** Results on neuropsychological tests

		*Mean* ± *SD*	*Number of patients with a deficit score (score under 2SD of the mean)*
*Purdue Pegboard test* *(z-score)*	Right hand	−1,88 ± 1,92 Min: −6,43 Max: 1,83	16
	Left hand	−1,21 ± 1,65 Min: −5,70 Max: 1,55	10
	Both hands	−2,05 ± 1,91 Min: −5,50 Max: 1,89	17
	Assembly	−1,60 ± 1,63 Min: −5,15 Max: 2,00	14
*ICARS* *(Score; a higher score implies worse performance)*	Total score	10,03 ± 9,94 Min: 0 Max: 42	19
	Kinetic function	4,40 ± 5,38 Min: 0 Max: 22	8
	Posture and gait	3,63 ± 3,57 Min: 0 Max: 15	4
	Oculomotor disorders	1,45 ± 1,26 Min: 0 Max: 5	0
	Speech	0,55 ± 1,11 Min: 0 Max: 4	0
*Wechsler intelligence scale (Standard Score; a higher score implies better performance)*	FSIQ	94,82 ± 16,10 Min: 56 Max: 128	4
	FRI	95,62 ± 14,14 Min: 63 Max: 128	1
	WMI	94,67 ± 16,54 Min: 56 Max: 136	3
	PSI	87,49 ± 13,70 Min: 59 Max: 114	5
	VCI	101,71 ± 18,71 Min: 61 Max: 137	2

### Lesion Characteristics

**Fig. 1:**
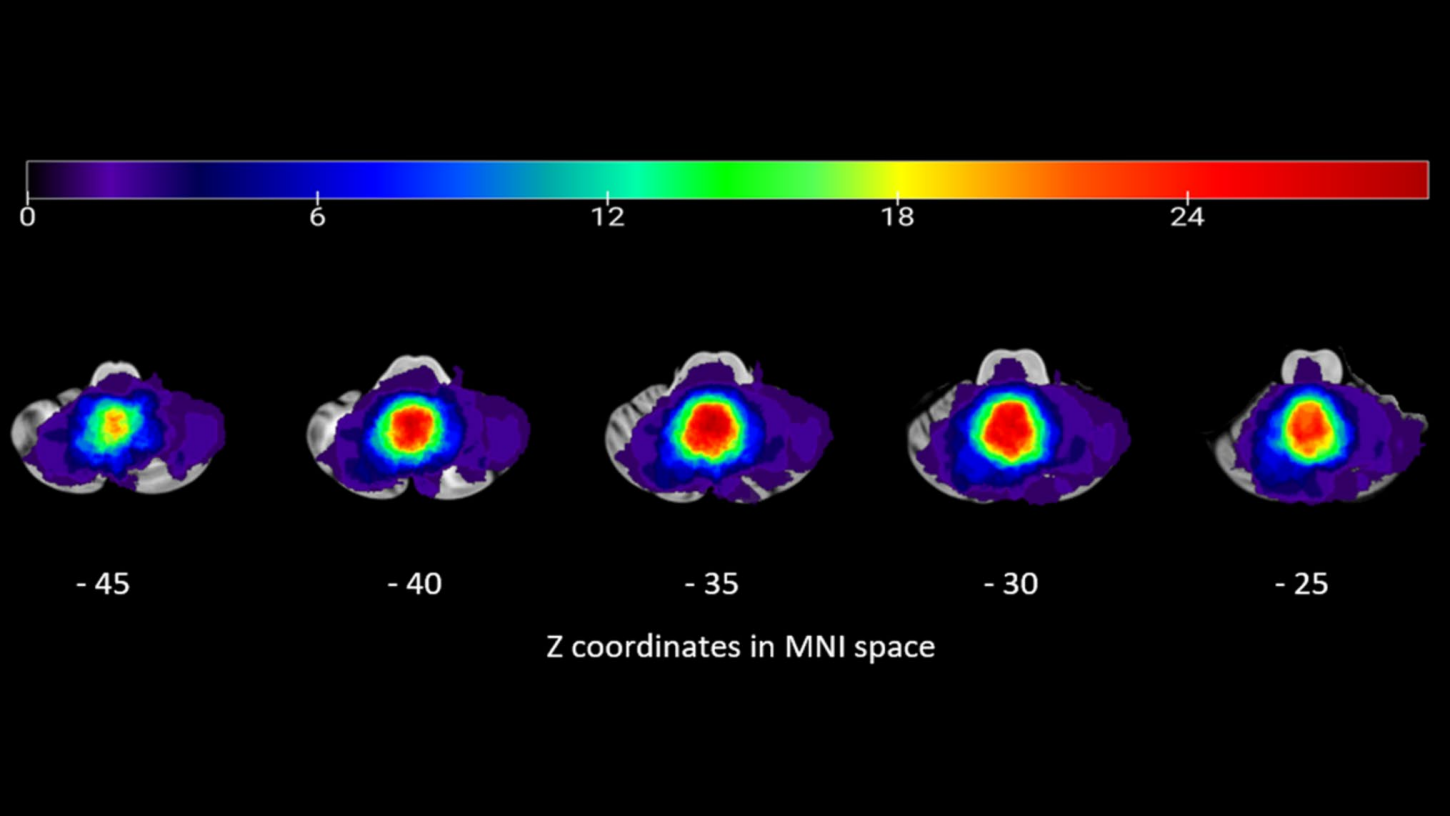
Lesion overlay in cerebellar tumor patients (n = 40). Color code indicates numbers of overlapping lesions. Numbers on the bottom of the cerebellum slices indicate the z coordinates (MNI) of each axial slice. The color indicates the number of patients with damage to a given voxel (from 0 to 29). The greatest lesion overlap (red) occurred in the regions of interest

Maximum overlap for all lesions of cerebellar tumor was seen in posterior vermis of the cerebellum (VIIIa, VIIIb, Crus II, VIIb, IX and VI), in left posterior cerebellum (Crus II and VIIb), and right posterior cerebellum (Crus II) (Fig. 1).

## Voxel Lesion Symptom Mapping (VLSM) Results

### Purdue Pegboard Test

**Fig. 2:**
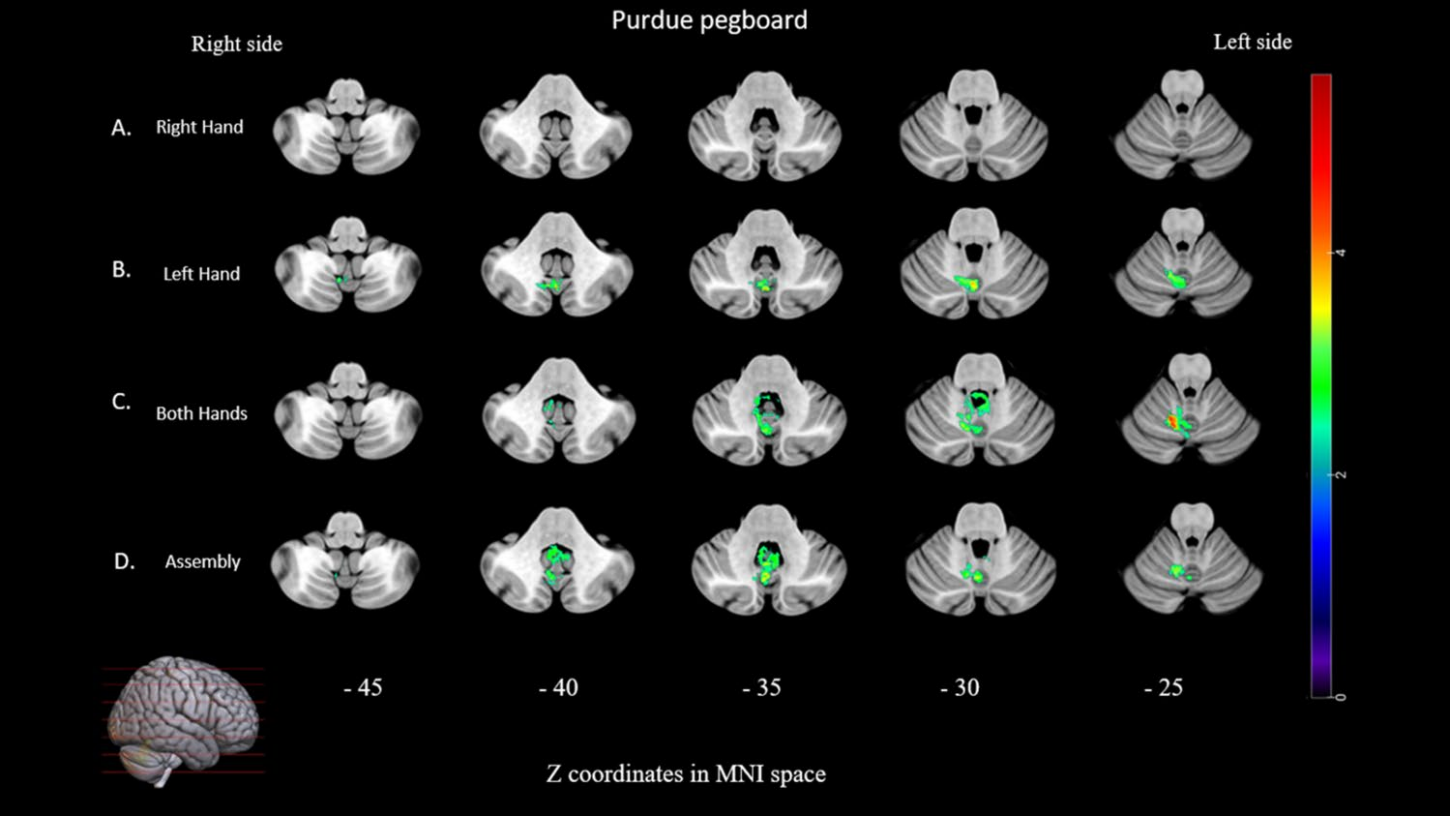
Lesion symptom mapping of motor and ataxia. For each test/subtests, voxel-based lesion system mapping compared voxel by voxel the performance of cerebellar tumor patients with a lesion against those without a lesion in a given voxel. Colored areas indicate a significant association between the presence of a lesion in that location and lower score for the Pegboard subtests (A, B, C, D), and a higher score for the ICARS (E) since higher scores are associated with higher impairment. The results are overlaid onto an MRI template brain in Montreal Neurological Institute space for visualization purposes. Color bar indicates Z scores. Numbers on the bottom of the cerebellum slices indicate the z coordinates (MNI) of each axial slice. Right-sided lesions being flipped to the left on cerebellar template

Right-hand fine motricity was not significantly associated with lesions to the cerebellum. Lesions to posterior vermis cerebellum (VI, Crus II, VIIb, VIIIa, VIIIb and IX) and anterior (V) (max volume = 2064 voxels, max T mean = 3.19), were associated to impaired left-hand fine motricity. Lesions to the posterior (VI, Crus II, VIIb, VIIIa, VIIIb, IX and X) and anterior (I-IV and V) (max volume = 3048 voxels, max T mean = 3.42) were associated with lower scores when both hands were used. Finally, lesions to the posterior cerebellum (VI, Crus II, VIIb, VIIIa, VIIIb, IX and X) and anterior (V) (max volume = 2962, max T mean = 3.08) were associated to the assembly subtest, that involves a more complex cognitive/planning task (see Fig. 2 and supplementary Tables 1—2).

### Ataxia Scale – ICARS

**Fig. 3:**
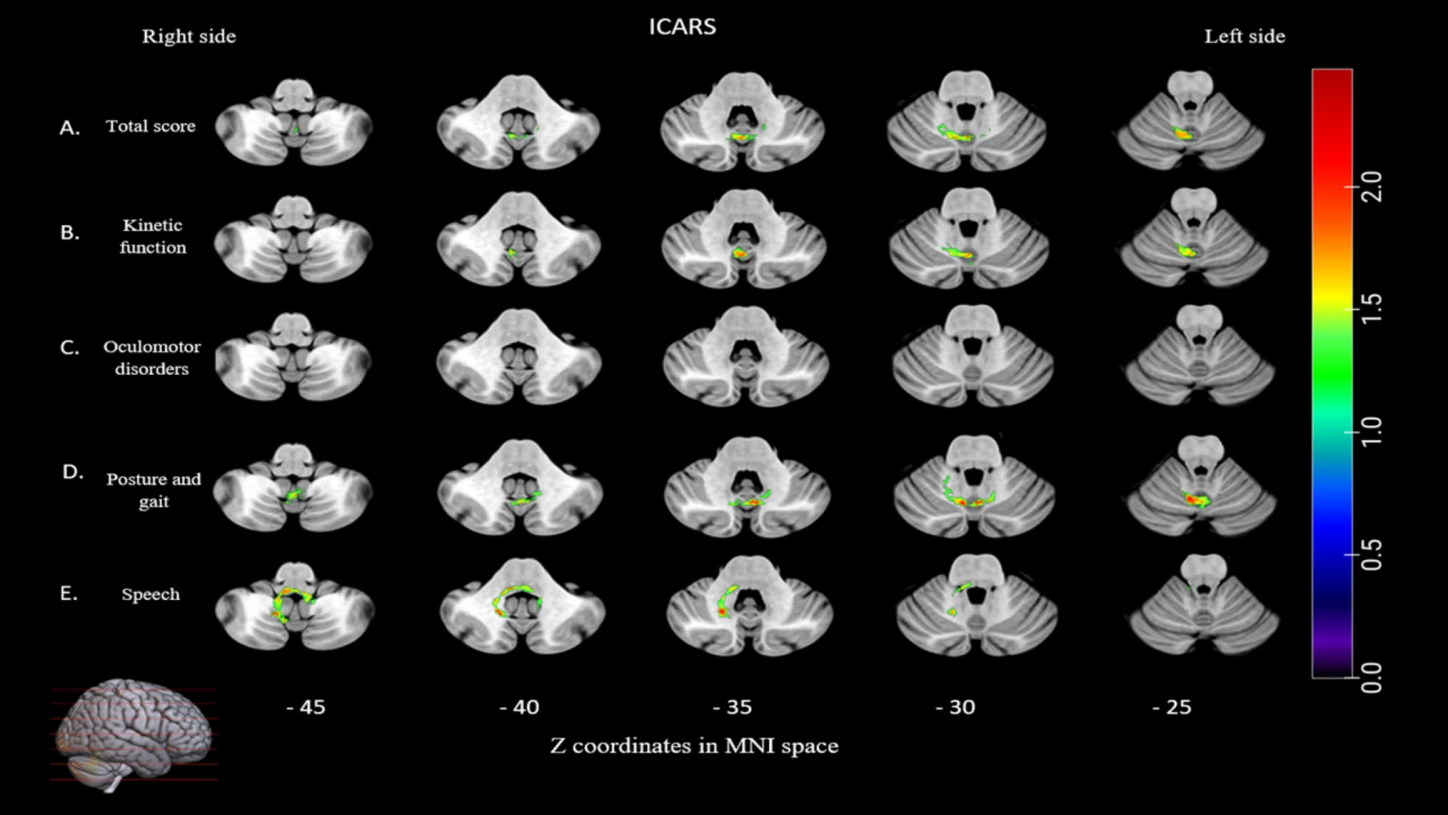
Lesion symptom mapping of ICARS. For each subscores, voxel-based lesion system mapping compared voxel by voxel the performance of cerebellar tumor patients with a lesion against those without a lesion in a given voxel. Colored areas indicate a significant association between the presence of a lesion in that location and higher score for ICARS subscore (which means worse performance). The results are overlaid into an MRI template brain in Montreal Neurological Institute space for visualization purposes. Color bar indicates Z scores. Numbers on the bottom of the cerebellum slices indicate the z coordinates (MNI) of each axial slice. Right-sided lesions being flipped to the left on cerebellar template

Lesions to the posterior cerebellum (VI,VIIb, VIIIa, VIIIb, and IX) and anterior (I-IV and V) (max volume = 1555 voxels, max T mean = 3.11) were also associated with higher scores to the total score subscore of the ICARS, and therefore a higher ataxia impairment (see Fig. 2 and supplementary Tables 1–2). Lesions to posterior (VI, Crus II, VIIb, VIIIa, VIIIb and IX) and anterior (I-IV and V) (max volume = 1354 voxels, max T mean = 2.96) were associated to kinetic function higher scores. Score to oculomotor disorders subscore were not significantly associate to lesions to the cerebellum. Lesions to posterior (VI, VIIb, VIIIa, VIIIb, and IX) and anterior (I-IV and V) (max volume = 2833 voxels, max T mean = 3.81) were associated with a higher posture and gait deficit subscore. Lesions to posterior (VIIIa, VIIIb and IX) and anterior (I-IV) (max volume = 1410 voxels, max T mean = 3.65) were associated with a higher speech deficit subscore (see Fig. 3 and supplementary Table 1—2).

Percentage of voxels in the anterior and posterior cerebellum showing a significant association between the presence of a lesion at that location and a lower score for purdue pegboard and higher score for the ataxia scale are summarized in Fig. 5.

### Intelligence – Wechsler Scale

**Fig. 4:**
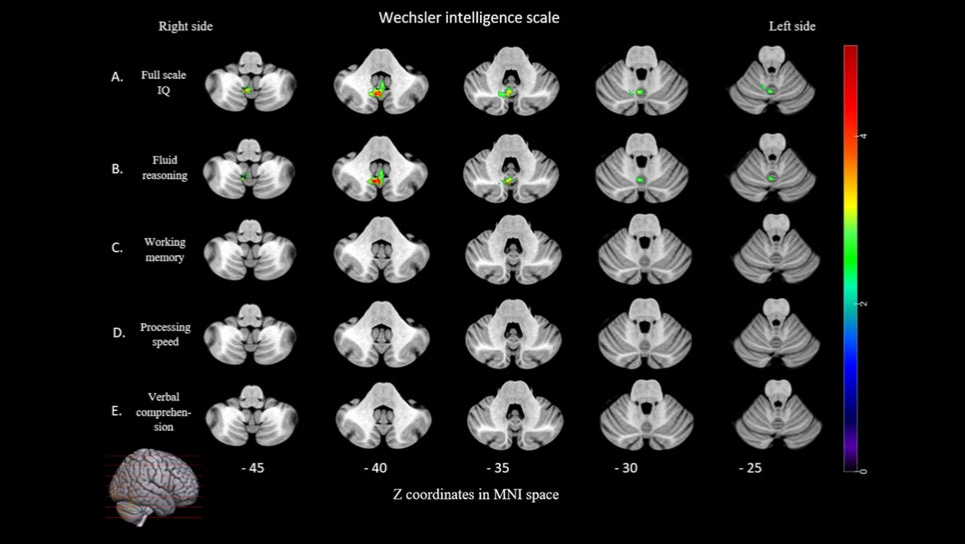
Lesion symptom mapping of intelligence scale. For each test/subtests, voxel-based lesion system mapping compared voxel by voxel the performance of cerebellar tumor patients with a lesion against those without a lesion in a given voxel. Colored areas indicate a significant association between the presence of a lesion in that location and lower score for the intelligence scales indexes. The results are overlaid into an MRI template brain in Montreal Neurological Institute space for visualization purposes. Color bar indicates Z scores. Numbers on the bottom of the cerebellum slices indicate the z coordinates (MNI) of each axial slice. Right-sided lesions being flipped to the left on cerebellar template

Lower scores at the full-scale intelligence were associated with lesions to the posterior cerebellum (VI, Crus I, Crus II, VIIb, VIIIa, VIIIb and IX) and the anterior cerebellum (V) (max volume = 2048 voxels, max T mean = 3.38). Lower scores at fluid reasoning were associated with lesions in the posterior (VI, Crus II, VIIb, VIIIa, VIIIb and IX) and anterior cerebellum (V) (max volume = 1618 voxels, max T mean = 4.40). No lesions in the cerebellum were significantly associated with impaired working memory, processing speed and verbal comprehension (see Fig. 4 and supplementary Tables 1—2**)**.

**Fig. 5:**
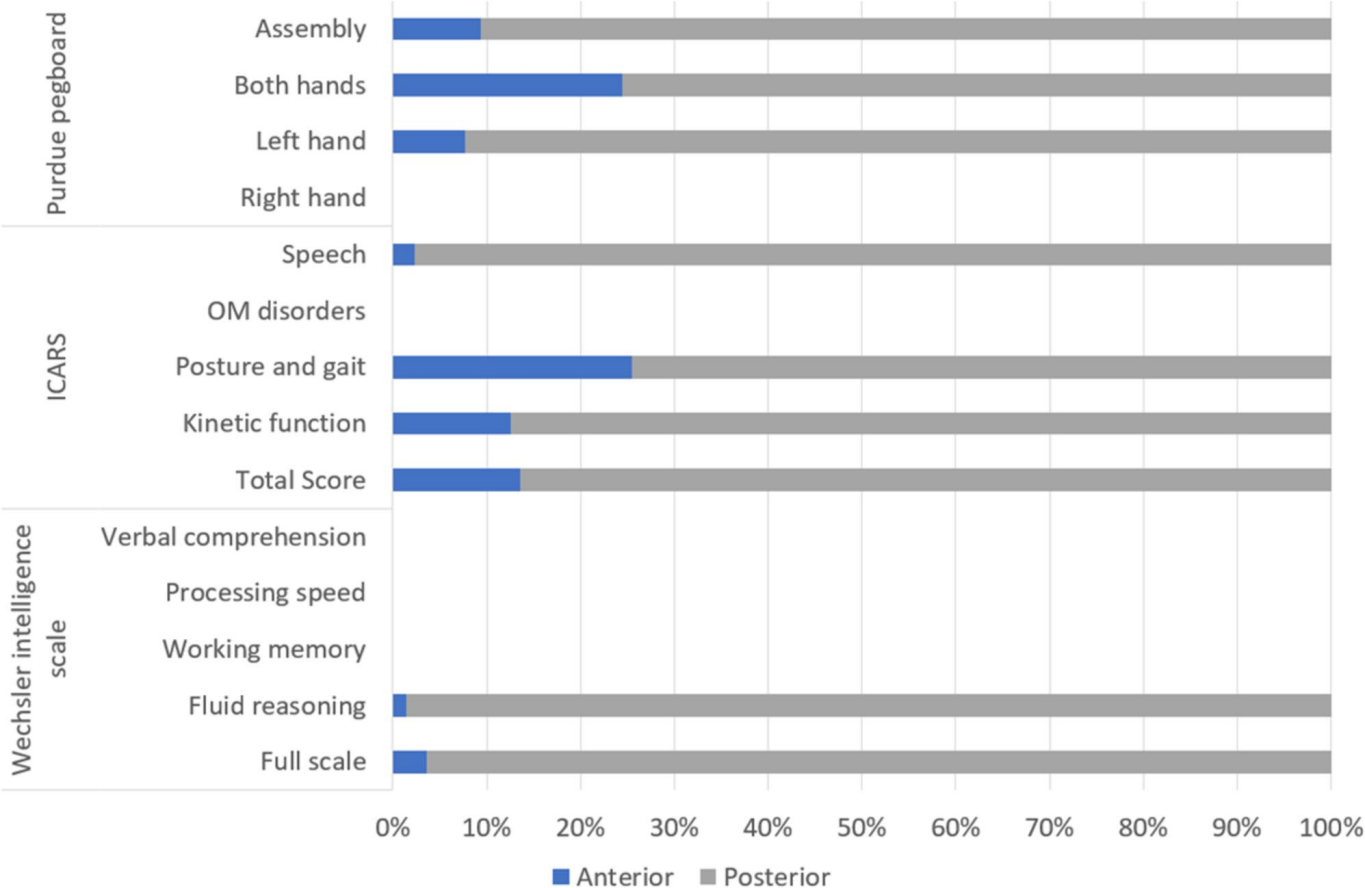
Percentage of voxels located in the anterior and posterior cerebellum showing a significant association between the presence of a lesion at that location and a deficit score at each tests/subtest

Percentage of voxels in the anterior and posterior cerebellum showing a significant association between the presence of a lesion at that location and a lower score for cognitive scale are summarized in Fig. 5.

## Discussion

In this study, we mapped multiple aspects of motricity and cognition in the cerebellum of children and teenager patients with lesions due to tumor resection while controlling for the factors known to affect motricity and cognition after a lesion.

Our first primary result is that lesions in both the anterior and posterior cerebellum led to motor deficits. This somewhat questions the model linking the anterior cerebellum lesions to motor deficits and the posterior cerebellum lesions to cognitive deficits that still permeate currently [46]. Indeed, while it is already acknowledged – even as part of the aforementioned model – that the posterior cerebellum also contains motor representations [46], these seem limited to specific posterior cerebellum regions (lobule VIII in [46]) therefore less reliably representing motor actions. At odds with this, recent evidence suggests that the posterior cerebellum can reliably be involved in motor control. Mottolese et al. [38] made use of direct electrical stimulation of the posterior cerebellum to elicit movements. They concluded that movements could be elicited via direct stimulation of the posterior cerebellum and that these movements were somatotopically mapped into the cerebellum (see also [12, 60] for similar conclusions using neuroimaging), apart from the intermediate regions of the posterior cerebellum (hemispheric lobules VIIb-IX) whose electrical stimulation less consistently resulted in movements. The current study extends these findings by revealing associations between lesions in multiple posterior cerebellum regions (Crus II, lobules VI, VIIb, VIIIa, VIIIb, IX, X) to motor deficits, along with anterior cerebellar regions. On these grounds, the first primary result suggests that anterior and posterior cerebellar regions equally contribute to motricity. It also supports a “motricity- and/or somatotopy-centered” view of the cerebellum’s function rather than a “motricity vs. cognition” localisationist view and confirms its crucial role in motricity.

Our second primary result is that lesions in the anterior cerebellum little affected cognitive performances: lesions in the anterior cerebellum consistently affected intellectual quotient, processing speed but to a minor extent (66 and 23 voxels in the anterior cerebellum, respectively, see Fig. 5). On the contrary, lesions in the posterior region of the cerebellum greatly impaired the patients’ cognition. From a theoretical perspective, this finding first comes in contradiction with the “somatotopic” view of the cerebellum’s function (see e.g. [12, 38, 60]) and rather supports the involvement of the cerebellum in cognition (see e.g. [47, 50]). However, to date, the most prominent model linking the cerebellum to cognition does not support either a motor or a cognitive view of the cerebellum’s function. Instead, it states that these do not perfectly co-localize in the cerebellum, with the anterior cerebellum consistently representing motor activations and the posterior part being more dedicated to cognitive processing [47]. Here, the current study allowed for the investigation of the effect of cerebellar lesions on multiple cognitive functions and our results suggest that both the anterior and the posterior cerebellum are involved in both motricity and cognition, but to different extents. This somewhat mitigates the distinction of the functional of the anterior vs. posterior cerebellum (e.g. see [32]), while supporting Schmahmann’s neuroanatomical model of the cerebellum’s involvement in cognition [46]. Secondly, this fuels the discussion of the possible intricate links between cognition and motricity in the cerebellum.

Of important note, this dichotomy is based on data collected on the entire cerebellum. In the current study however, most lesions were located in the vermal regions (see Fig. 1), as most of the pediatric cerebellar tumors occur in the cerebellar midline [34, 41]. Actually, medial vs. lateral functional differences have been previously highlighted beyond the anterior vs. posterior dichotomy (e.g. see [29]). As a consequence, the current results do not allow to generalize our findings to more lateral regions of the cerebellum. Therefore, both the current results and additional accounts of the functional organization of the cerebellum (e.g. [29]) claim for a more complex schema of the fine-grained cerebellum functional organization.

It is noteworthy that current and past experimental studies cannot specifically address the question of the local computations occurring at the level of the cerebellum because they cannot dissociate the cerebellum’s function from the network(s) it belongs to. In addition, connectivity studies have described multiple functional links between regions of the cerebellum and so-called “eloquent” (i.e. known to be highly specialized for a specific function) regions. For example, analyses of resting functional connectivity between Broca’s area and the cerebellum have revealed a decoupling between the latter two regions in patients with autistic syndrome disorder [66], whose language function is altered. Similarly, meta-analytic effective connectivity analyses between Broca’s area and language-aspecific areas suggest that modulation of directional connectivity between Broca’s area and the cerebellum occurs during language preparation [19]. Analogous findings have suggested that functional connectivity patterns between the cerebellum and the dorsal attention network are at play during attentional tasks [13, 26, 30], social cognition processes [62, 63], and executive control [26].

From this perspective, associations between cerebellum functioning and cognition should be cautiously taken because of the involvement of the cerebellum within multiple functional brain networks. It also leaves room for the proposition of alternative accounts of the cerebellum’s function during mental processes beyond the motricity vs. cognition dichotomy. Indeed, recent research [26, 49, 70] has proposed a predictive role for the cerebellum in cognition based on the “forward model” linking sensory prediction to actual motor output (“efferent copies”) in the context of motricity [49]. In that case, in addition to the fact that a deficient “forward model” in the cerebellum would lead to gross observable motor deficits due to prediction deficits, it would also lead to more subtle deficits in the cognitive domain [49, 62, 63]. On these grounds, a general “prediction” function of the cerebellum would fit both the cognitive and motor views of this structure and explain how lesions in any cerebellar area can cause both motor and cognitive difficulties. Future effort research efforts should be put into understanding the fine-grained overlapping mechanisms/computations underlying prediction in motricity and cognition in the cerebellum.

We also put a note of caution regarding virtually any study on the role of the cerebellum on cognition vs. motricity. Testing cognitive functions most of the time also requires the recruitment of motor components (e.g. finger-response, language, drawing or writing), and conversely (see for example the use of the pegboard to predict cognitive deficits in [11]), which highly challenges the disentangling of effects on cognitive vs. motor changes. On the contrary, there exist tests that allow the dissociation of the two latter effects (e.g. cognitive vs. motor component of the Stroop test responses in [27]). Alternatively, future tests could take advantage of measures of brain signals (e.g. event-related potentials) as measures of cognitive components (e.g. “N2pc” and “PD” for different aspects of attentional processes, see e.g. [43]) to bypass the noise generated by motor actions during cognitive processes (and see [32], who varied the type of motor demand across task conditions as part of an fMRI study).

In summary, in this study, we used VLSM to causally investigate the involvement of the cerebellum in motricity and cognition. Our results showed that both motor and cognitive components were affected by posterior cerebellar lesions, while primarily motor deficits were caused by lesions in the anterior cerebellum. They suggest that the spatial dichotomy between the “cognitive” and the “motor” cerebellum (e.g. [47]) partially holds but is not stringent. Models integrating the involvement of the cerebellum in both motricity and cognition (e.g. a predictive function, see [49, 70]) may better fit the actual fine-grained computations occurring in the cerebellum.

## Supplementary Information

Below is the link to the electronic supplementary material.

Supplementary file1 (DOCX 287 KB)

## Data Availability

Data can be available upon request to the corresponding authors.
