# Supplementary material for: Causally Mapping the Cerebellum in Children and Young Adults: from Motor to Cognition
Source: Cerebellum. 2025 Jan 16;24(2):31. doi: 10.1007/s12311-024-01778-8 (PMC11735598; doi:10.1007/s12311-024-01778-8)
Supplement: Supplementary file 1 — Supplementary file1 (DOCX 287 KB) [file 12311_2024_1778_MOESM1_ESM.docx]

**Table 1.** VLSM results for motor tests.

|  |  |  | **Peak MNI coordinate** | | | **Center MNI coordinate** | | |  | Localisation | | |
| --- | --- | --- | --- | --- | --- | --- | --- | --- | --- | --- | --- | --- |
|  | **Cluster size** | **T mean** | **x** | **y** | **z** | **x2** | **y2** | **z2** | **number of voxels in the cluster** | Side | Lobe | Area |
| Right hand with all covariates |  |  |  |  |  |  |  |  |  |  |  |  |
| Right hand without age at surgery as covariate |  |  |  |  |  |  |  |  |  |  |  |  |
| Right hand without deep nuclei as covariate |  |  |  |  |  |  |  |  |  |  |  |  |
| Right hand without gender as covariate |  |  |  |  |  |  |  |  |  |  |  |  |
| Right hand without treatment as covariate |  |  |  |  |  |  |  |  |  |  |  |  |
| Right hand without any covariate |  |  |  |  |  |  |  |  |  |  |  |  |
| Left hand with all covariates | 2064 | 2.72 | 0.00 | -64.00 | -20.00 | -0.23 | -63.77 | -19.46 | 4 | Anterior | Left | V |
|  | 2064 | 3.05 | 9.00 | -62.00 | -25.00 | 6.08 | -61.54 | -21.94 | 142 | Anterior | Right | V |
|  | 2064 | 2.95 | -2.00 | -66.00 | -27.00 | 1.92 | -66.62 | -25.64 | 557 | Posterior | Vermis | VI |
|  | 2064 | 2.94 | 7.00 | -66.00 | -30.00 | 9.09 | -64.89 | -25.83 | 63 | Posterior | Right | VI |
|  | 2064 | 3.16 | 2.00 | -72.00 | -36.00 | 1.29 | -72.06 | -32.68 | 128 | Posterior | Vermis | CrusII |
|  | 2064 | 2.68 | 4.00 | -73.00 | -39.00 | 5.61 | -74.15 | -33.50 | 4 | Posterior | Right | CrusII |
|  | 2064 | 2.75 | -5.00 | -70.00 | -39.00 | -4.93 | -70.22 | -36.14 | 14 | Posterior | Left | VIIb |
|  | 2064 | 3.19 | 2.00 | -67.00 | -31.00 | 1.57 | -68.34 | -31.33 | 194 | Posterior | Vermis | VIIb |
|  | 2064 | 2.80 | 7.00 | -68.00 | -33.00 | 9.31 | -68.55 | -37.16 | 88 | Posterior | Right | VIIb |
|  | 2064 | 2.57 | -7.00 | -67.00 | -38.00 | -6.80 | -66.01 | -35.63 | 5 | Posterior | Left | VIIIa |
|  | 2064 | 3.06 | -2.00 | -71.00 | -37.00 | 1.11 | -67.56 | -36.88 | 555 | Posterior | Vermis | VIIIa |
|  | 2064 | 2.81 | 8.00 | -65.00 | -32.00 | 9.28 | -66.36 | -37.83 | 27 | Posterior | Right | VIIIa |
|  | 2064 | 2.75 | 7.00 | -66.00 | -45.00 | 1.79 | -64.12 | -41.76 | 120 | Posterior | Vermis | VIIIb |
|  | 2064 | 2.83 | 8.00 | -65.00 | -46.00 | 7.43 | -64.41 | -46.00 | 5 | Posterior | Right | VIIIb |
|  | 2064 | 2.52 | 2.00 | -62.00 | -46.00 | 1.16 | -61.66 | -45.68 | 6 | Posterior | Vermis | IX |
|  | 2064 | 2.66 | 6.00 | -61.00 | -42.00 | 6.32 | -62.30 | -44.28 | 3 | Posterior | Right | IX |
|  | 2064 | 2.69 | -8.00 | -64.00 | -34.00 | -8.00 | -64.34 | -34.66 | 3 | DN | Left | Dentate |
|  | 2064 | 2.89 | 10.00 | -63.00 | -29.00 | 11.53 | -64.37 | -30.73 | 139 | DN | Right | Dentate |
|  | 2064 | 2.68 | 10.00 | -58.00 | -25.00 | 8.88 | -60.22 | -27.64 | 7 | DN | Right | Interposed |
| Left hand without age at surgery as covariate | 2054 | 2.76 | 0.00 | -64.00 | -20.00 | -0.22 | -63.78 | -19.46 | 4 | Anterior | Left | V |
|  | 2054 | 3.09 | 9.00 | -62.00 | -25.00 | 6.08 | -61.53 | -21.95 | 143 | Anterior | Right | V |
|  | 2054 | 2.47 | -5.00 | -70.00 | -29.00 | -5.00 | -69.50 | -28.00 | 2 | Posterior | Left | VI |
|  | 2054 | 2.99 | -2.00 | -66.00 | -27.00 | 1.90 | -66.64 | -25.64 | 563 | Posterior | Vermis | VI |
|  | 2054 | 2.96 | 7.00 | -66.00 | -30.00 | 9.07 | -64.97 | -25.87 | 64 | Posterior | Right | VI |
|  | 2054 | 2.47 | -5.00 | -71.00 | -33.00 | -5.00 | -71.00 | -33.00 | 1 | Posterior | Left | CrusII |
|  | 2054 | 3.19 | 2.00 | -72.00 | -36.00 | 1.30 | -72.05 | -32.68 | 129 | Posterior | Vermis | CrusII |
|  | 2054 | 2.71 | 4.00 | -73.00 | -39.00 | 5.61 | -74.15 | -33.50 | 4 | Posterior | Right | CrusII |
|  | 2054 | 2.71 | -5.00 | -70.00 | -39.00 | -4.94 | -70.23 | -36.14 | 14 | Posterior | Left | VIIb |
|  | 2054 | 3.21 | 2.00 | -67.00 | -31.00 | 1.44 | -68.40 | -31.33 | 202 | Posterior | Vermis | VIIb |
|  | 2054 | 2.81 | 7.00 | -68.00 | -33.00 | 9.27 | -68.55 | -37.07 | 90 | Posterior | Right | VIIb |
|  | 2054 | 2.57 | -7.00 | -67.00 | -38.00 | -6.76 | -66.26 | -35.78 | 4 | Posterior | Left | VIIIa |
|  | 2054 | 3.05 | -2.00 | -71.00 | -37.00 | 1.07 | -67.50 | -36.75 | 574 | Posterior | Vermis | VIIIa |
|  | 2054 | 2.78 | 8.00 | -65.00 | -32.00 | 9.45 | -66.49 | -38.67 | 31 | Posterior | Right | VIIIa |
|  | 2054 | 2.78 | 1.00 | -64.00 | -38.00 | 2.02 | -63.99 | -40.86 | 96 | Posterior | Vermis | VIIIb |
|  | 2054 | 2.95 | 8.00 | -65.00 | -46.00 | 7.66 | -64.37 | -46.00 | 3 | Posterior | Right | VIIIb |
|  | 2054 | 2.71 | 6.00 | -61.00 | -42.00 | 6.48 | -61.95 | -43.43 | 2 | Posterior | Right | IX |
|  | 2054 | 2.68 | -8.00 | -64.00 | -34.00 | -8.00 | -64.34 | -34.66 | 3 | DN | Left | Dentate |
|  | 2054 | 2.92 | 10.00 | -63.00 | -29.00 | 11.26 | -64.22 | -30.56 | 118 | DN | Right | Dentate |
|  | 2054 | 2.73 | 10.00 | -58.00 | -25.00 | 8.88 | -60.22 | -27.64 | 7 | DN | Right | Interposed |
| Left hand without deep nuclei as covariate |  |  |  |  |  |  |  |  |  |  |  |  |
| Left hand without gender as covariate | 1927 | 2.55 | 0.00 | -64.00 | -20.00 | -0.60 | -62.51 | -20.72 | 13 | Anterior | Left | V |
|  | 1927 | 2.99 | 9.00 | -62.00 | -25.00 | 5.70 | -61.38 | -21.85 | 168 | Anterior | Right | V |
|  | 1927 | 2.99 | -2.00 | -66.00 | -27.00 | 1.65 | -66.00 | -25.50 | 495 | Posterior | Vermis | VI |
|  | 1927 | 3.02 | 13.00 | -64.00 | -27.00 | 9.38 | -63.79 | -25.39 | 48 | Posterior | Right | VI |
|  | 1927 | 3.06 | 0.00 | -72.00 | -36.00 | 0.61 | -71.83 | -33.22 | 91 | Posterior | Vermis | CrusII |
|  | 1927 | 3.02 | 4.00 | -73.00 | -39.00 | 4.00 | -73.00 | -39.00 | 1 | Posterior | Right | CrusII |
|  | 1927 | 2.74 | -5.00 | -70.00 | -39.00 | -4.94 | -70.22 | -36.16 | 14 | Posterior | Left | VIIb |
|  | 1927 | 3.21 | 2.00 | -67.00 | -31.00 | 1.40 | -68.24 | -31.31 | 184 | Posterior | Vermis | VIIb |
|  | 1927 | 2.85 | 7.00 | -68.00 | -33.00 | 8.41 | -68.40 | -36.08 | 55 | Posterior | Right | VIIb |
|  | 1927 | 2.56 | -7.00 | -67.00 | -38.00 | -6.80 | -66.01 | -35.62 | 5 | Posterior | Left | VIIIa |
|  | 1927 | 3.00 | -2.00 | -71.00 | -37.00 | 0.79 | -67.33 | -36.52 | 562 | Posterior | Vermis | VIIIa |
|  | 1927 | 2.77 | 8.00 | -65.00 | -32.00 | 8.98 | -66.27 | -37.45 | 23 | Posterior | Right | VIIIa |
|  | 1927 | 2.69 | 1.00 | -64.00 | -38.00 | 1.22 | -63.96 | -41.37 | 132 | Posterior | Vermis | VIIIb |
|  | 1927 | 2.82 | 8.00 | -65.00 | -46.00 | 7.08 | -64.66 | -46.29 | 3 | Posterior | Right | VIIIb |
|  | 1927 | 2.54 | 0.00 | -61.00 | -46.00 | 1.00 | -61.57 | -45.17 | 7 | Posterior | Vermis | IX |
|  | 1927 | 2.53 | 6.00 | -61.00 | -42.00 | 6.00 | -61.00 | -42.00 | 1 | Posterior | Right | IX |
|  | 1927 | 2.73 | -8.00 | -64.00 | -34.00 | -8.00 | -64.33 | -34.65 | 3 | DN | Left | Dentate |
|  | 1927 | 2.95 | 10.00 | -63.00 | -29.00 | 11.19 | -64.09 | -30.32 | 114 | DN | Right | Dentate |
|  | 1927 | 2.67 | 10.00 | -58.00 | -25.00 | 8.66 | -60.44 | -28.04 | 8 | DN | Right | Interposed |
| Left hand without treatment as covariate |  |  |  |  |  |  |  |  |  |  |  |  |
| Left hand without any covariate |  |  |  |  |  |  |  |  |  |  |  |  |
| Both hands with all covariates | 3048 | 3.06 | 0.00 | -52.00 | -28.00 | -1.50 | -49.06 | -27.37 | 16 | Anterior | Left | I_IV |
|  | 3048 | 2.79 | 11.00 | -52.00 | -21.00 | 7.57 | -50.60 | -20.47 | 90 | Anterior | Right | I_IV |
|  | 3048 | 2.51 | 0.00 | -65.00 | -18.00 | 0.00 | -61.01 | -19.20 | 5 | Anterior | Left | V |
|  | 3048 | 3.42 | 15.00 | -58.00 | -26.00 | 9.83 | -59.02 | -21.30 | 551 | Anterior | Right | V |
|  | 3048 | 2.75 | 6.00 | -63.00 | -27.00 | 2.44 | -66.69 | -24.74 | 424 | Posterior | Vermis | VI |
|  | 3048 | 3.28 | 13.00 | -64.00 | -26.00 | 12.25 | -64.06 | -24.97 | 336 | Posterior | Right | VI |
|  | 3048 | 2.78 | 1.00 | -71.00 | -33.00 | 0.81 | -71.36 | -33.24 | 45 | Posterior | Vermis | CrusII |
|  | 3048 | 2.84 | 2.00 | -70.00 | -33.00 | 1.72 | -68.03 | -31.08 | 127 | Posterior | Vermis | VIIb |
|  | 3048 | 2.90 | 8.00 | -67.00 | -31.00 | 7.50 | -67.10 | -32.02 | 18 | Posterior | Right | VIIb |
|  | 3048 | 2.85 | 0.00 | -71.00 | -36.00 | 2.09 | -66.45 | -35.31 | 324 | Posterior | Vermis | VIIIa |
|  | 3048 | 2.91 | 8.00 | -65.00 | -32.00 | 8.16 | -64.51 | -32.63 | 6 | Posterior | Right | VIIIa |
|  | 3048 | 2.69 | 7.00 | -61.00 | -39.00 | 4.84 | -61.76 | -37.51 | 89 | Posterior | Vermis | VIIIb |
|  | 3048 | 2.94 | 8.00 | -61.00 | -37.00 | 8.00 | -61.00 | -37.00 | 1 | Posterior | Right | VIIIb |
|  | 3048 | 2.63 | -5.00 | -53.00 | -32.00 | -5.50 | -51.89 | -34.96 | 52 | Posterior | Left | IX |
|  | 3048 | 2.61 | 5.00 | -57.00 | -33.00 | 0.75 | -55.06 | -34.03 | 173 | Posterior | Vermis | IX |
|  | 3048 | 2.72 | 8.00 | -57.00 | -35.00 | 8.21 | -51.92 | -37.01 | 349 | Posterior | Right | IX |
|  | 3048 | 2.71 | -1.00 | -51.00 | -30.00 | 0.44 | -48.52 | -34.80 | 99 | Posterior | Vermis | X |
|  | 3048 | 2.91 | 9.00 | -66.00 | -31.00 | 12.47 | -61.80 | -29.84 | 175 | DN | Right | Dentate |
|  | 3048 | 2.50 | -6.00 | -59.00 | -34.00 | -6.50 | -57.01 | -31.64 | 8 | DN | Left | Interposed |
|  | 3048 | 2.90 | 10.00 | -58.00 | -25.00 | 8.07 | -57.94 | -29.45 | 154 | DN | Right | Interposed |
|  | 3048 | 2.69 | -3.00 | -52.00 | -28.00 | -2.38 | -53.54 | -28.62 | 3 | DN | Left | Fastigial |
|  | 3048 | 2.88 | 4.00 | -54.00 | -29.00 | 4.33 | -53.00 | -28.67 | 3 | DN | Right | Fastigial |
| Both hands without age at surgery as covariate | 2991 | 3.08 | 0.00 | -51.00 | -28.00 | -1.50 | -49.06 | -27.37 | 16 | Anterior | Left | I_IV |
|  | 2991 | 2.75 | 11.00 | -52.00 | -21.00 | 7.45 | -50.66 | -20.48 | 85 | Anterior | Right | I_IV |
|  | 2991 | 2.54 | 0.00 | -65.00 | -18.00 | 0.00 | -61.01 | -19.20 | 5 | Anterior | Left | V |
|  | 2991 | 3.33 | 13.00 | -60.00 | -24.00 | 9.72 | -59.07 | -21.31 | 549 | Anterior | Right | V |
|  | 2991 | 2.78 | 6.00 | -63.00 | -27.00 | 2.46 | -66.48 | -24.76 | 418 | Posterior | Vermis | VI |
|  | 2991 | 3.28 | 11.00 | -63.00 | -22.00 | 11.16 | -64.48 | -24.55 | 267 | Posterior | Right | VI |
|  | 2991 | 2.79 | 1.00 | -71.00 | -33.00 | 0.51 | -71.31 | -33.39 | 44 | Posterior | Vermis | CrusII |
|  | 2991 | 2.85 | 2.00 | -70.00 | -33.00 | 1.66 | -68.07 | -31.14 | 129 | Posterior | Vermis | VIIb |
|  | 2991 | 2.86 | 7.00 | -68.00 | -33.00 | 7.45 | -67.06 | -31.92 | 16 | Posterior | Right | VIIb |
|  | 2991 | 2.87 | 0.00 | -71.00 | -36.00 | 2.09 | -66.43 | -35.30 | 327 | Posterior | Vermis | VIIIa |
|  | 2991 | 2.87 | 8.00 | -65.00 | -32.00 | 8.16 | -64.49 | -32.62 | 6 | Posterior | Right | VIIIa |
|  | 2991 | 2.71 | 7.00 | -61.00 | -39.00 | 4.84 | -61.76 | -37.50 | 89 | Posterior | Vermis | VIIIb |
|  | 2991 | 2.97 | 8.00 | -61.00 | -37.00 | 8.00 | -61.00 | -37.00 | 1 | Posterior | Right | VIIIb |
|  | 2991 | 2.64 | -5.00 | -53.00 | -32.00 | -5.49 | -51.89 | -34.97 | 52 | Posterior | Left | IX |
|  | 2991 | 2.63 | 5.00 | -57.00 | -33.00 | 0.87 | -54.95 | -34.38 | 190 | Posterior | Vermis | IX |
|  | 2991 | 2.74 | 8.00 | -57.00 | -35.00 | 8.22 | -51.86 | -37.05 | 353 | Posterior | Right | IX |
|  | 2991 | 2.68 | -1.00 | -51.00 | -30.00 | 0.41 | -48.57 | -34.25 | 125 | Posterior | Vermis | X |
|  | 2991 | 2.83 | 9.00 | -66.00 | -31.00 | 12.11 | -62.14 | -29.78 | 143 | DN | Right | Dentate |
|  | 2991 | 2.48 | -6.00 | -59.00 | -34.00 | -5.81 | -57.10 | -31.31 | 20 | DN | Left | Interposed |
|  | 2991 | 2.92 | 10.00 | -58.00 | -25.00 | 8.03 | -57.91 | -29.43 | 150 | DN | Right | Interposed |
|  | 2991 | 2.70 | -3.00 | -52.00 | -28.00 | -2.38 | -53.54 | -28.62 | 3 | DN | Left | Fastigial |
|  | 2991 | 2.92 | 4.00 | -54.00 | -29.00 | 4.33 | -53.00 | -28.67 | 3 | DN | Right | Fastigial |
| Both hands without deep nuclei as covariate | 1864 | 2.87 | 0.00 | -51.00 | -28.00 | -1.49 | -49.07 | -27.37 | 16 | Anterior | Left | I_IV |
|  | 1864 | 2.79 | 11.00 | -52.00 | -21.00 | 8.06 | -50.36 | -20.10 | 68 | Anterior | Right | I_IV |
|  | 1864 | 3.32 | 15.00 | -58.00 | -26.00 | 10.52 | -58.75 | -21.29 | 467 | Anterior | Right | V |
|  | 1864 | 2.63 | 4.00 | -69.00 | -22.00 | 2.66 | -67.06 | -24.14 | 183 | Posterior | Vermis | VI |
|  | 1864 | 3.19 | 13.00 | -64.00 | -26.00 | 12.69 | -63.94 | -25.02 | 307 | Posterior | Right | VI |
|  | 1864 | 2.77 | 1.00 | -71.00 | -33.00 | 0.87 | -71.45 | -33.36 | 38 | Posterior | Vermis | CrusII |
|  | 1864 | 2.71 | 2.00 | -70.00 | -33.00 | 1.73 | -68.29 | -31.14 | 70 | Posterior | Vermis | VIIb |
|  | 1864 | 2.64 | 8.00 | -67.00 | -31.00 | 7.57 | -67.00 | -31.88 | 16 | Posterior | Right | VIIb |
|  | 1864 | 2.76 | 0.00 | -71.00 | -36.00 | 2.04 | -67.21 | -35.43 | 184 | Posterior | Vermis | VIIIa |
|  | 1864 | 2.77 | 8.00 | -65.00 | -32.00 | 8.32 | -64.98 | -32.32 | 3 | Posterior | Right | VIIIa |
|  | 1864 | 2.53 | 2.00 | -64.00 | -38.00 | 2.70 | -63.00 | -37.58 | 7 | Posterior | Vermis | VIIIb |
|  | 1864 | 2.56 | -5.00 | -53.00 | -32.00 | -5.98 | -50.57 | -34.78 | 31 | Posterior | Left | IX |
|  | 1864 | 2.55 | 5.00 | -57.00 | -33.00 | 1.23 | -54.84 | -34.40 | 64 | Posterior | Vermis | IX |
|  | 1864 | 2.63 | 8.00 | -57.00 | -35.00 | 7.83 | -50.76 | -37.14 | 202 | Posterior | Right | IX |
|  | 1864 | 2.61 | -1.00 | -51.00 | -30.00 | 0.96 | -48.09 | -34.77 | 74 | Posterior | Vermis | X |
|  | 1864 | 2.88 | 14.00 | -62.00 | -28.00 | 12.17 | -63.38 | -29.69 | 109 | DN | Right | Dentate |
|  | 1864 | 2.63 | 10.00 | -58.00 | -25.00 | 8.77 | -58.67 | -29.89 | 22 | DN | Right | Interposed |
|  | 1864 | 2.80 | -3.00 | -52.00 | -28.00 | -3.00 | -52.00 | -28.00 | 1 | DN | Left | Fastigial |
|  | 1864 | 2.44 | 4.00 | -53.00 | -29.00 | 4.50 | -52.50 | -28.50 | 2 | DN | Right | Fastigial |
| Both hands without gender as covariate | 3093 | 2.87 | 0.00 | -52.00 | -28.00 | -1.84 | -47.27 | -25.44 | 33 | Anterior | Left | I_IV |
|  | 3093 | 2.73 | 6.00 | -53.00 | -21.00 | 6.50 | -49.46 | -20.87 | 104 | Anterior | Right | I_IV |
|  | 3093 | 2.49 | 0.00 | -65.00 | -18.00 | -0.49 | -61.56 | -20.55 | 14 | Anterior | Left | V |
|  | 3093 | 3.39 | 15.00 | -58.00 | -26.00 | 9.57 | -59.10 | -21.30 | 579 | Anterior | Right | V |
|  | 3093 | 2.74 | 6.00 | -63.00 | -27.00 | 2.31 | -66.03 | -24.67 | 395 | Posterior | Vermis | VI |
|  | 3093 | 3.26 | 13.00 | -64.00 | -26.00 | 12.14 | -64.07 | -24.85 | 320 | Posterior | Right | VI |
|  | 3093 | 2.72 | 1.00 | -71.00 | -33.00 | 0.50 | -71.30 | -33.51 | 31 | Posterior | Vermis | CrusII |
|  | 3093 | 2.83 | 2.00 | -70.00 | -33.00 | 1.57 | -67.98 | -31.09 | 129 | Posterior | Vermis | VIIb |
|  | 3093 | 2.87 | 7.00 | -68.00 | -33.00 | 7.53 | -66.99 | -31.98 | 17 | Posterior | Right | VIIb |
|  | 3093 | 2.87 | 0.00 | -71.00 | -36.00 | 2.09 | -66.25 | -35.11 | 322 | Posterior | Vermis | VIIIa |
|  | 3093 | 2.86 | 8.00 | -65.00 | -32.00 | 8.17 | -64.51 | -32.63 | 6 | Posterior | Right | VIIIa |
|  | 3093 | 2.73 | 7.00 | -61.00 | -39.00 | 4.83 | -61.77 | -37.50 | 91 | Posterior | Vermis | VIIIb |
|  | 3093 | 3.01 | 8.00 | -61.00 | -37.00 | 8.00 | -61.00 | -37.00 | 1 | Posterior | Right | VIIIb |
|  | 3093 | 2.69 | -5.00 | -53.00 | -32.00 | -5.96 | -52.10 | -34.38 | 47 | Posterior | Left | IX |
|  | 3093 | 2.66 | 5.00 | -57.00 | -33.00 | 0.98 | -55.17 | -34.22 | 177 | Posterior | Vermis | IX |
|  | 3093 | 2.70 | 8.00 | -57.00 | -35.00 | 8.19 | -51.92 | -37.00 | 347 | Posterior | Right | IX |
|  | 3093 | 2.73 | -1.00 | -51.00 | -30.00 | 0.43 | -48.46 | -34.19 | 104 | Posterior | Vermis | X |
|  | 3093 | 2.88 | 9.00 | -66.00 | -31.00 | 12.21 | -61.93 | -29.70 | 171 | DN | Right | Dentate |
|  | 3093 | 2.52 | -6.00 | -59.00 | -34.00 | -5.82 | -57.18 | -31.24 | 22 | DN | Left | Interposed |
|  | 3093 | 2.91 | 10.00 | -58.00 | -25.00 | 8.10 | -57.64 | -29.33 | 177 | DN | Right | Interposed |
|  | 3093 | 2.76 | -3.00 | -52.00 | -28.00 | -2.38 | -53.54 | -28.62 | 3 | DN | Left | Fastigial |
|  | 3093 | 2.97 | 4.00 | -54.00 | -29.00 | 4.33 | -53.00 | -28.67 | 3 | DN | Right | Fastigial |
| Both hands without treatment as covariate | 2818 | 3.00 | 0.00 | -51.00 | -28.00 | -1.50 | -49.06 | -27.37 | 16 | Anterior | Left | I_IV |
|  | 2818 | 2.81 | 11.00 | -52.00 | -21.00 | 7.57 | -50.57 | -20.44 | 92 | Anterior | Right | I_IV |
|  | 2818 | 2.52 | 0.00 | -61.00 | -20.00 | 0.00 | -60.97 | -19.21 | 5 | Anterior | Left | V |
|  | 2818 | 3.23 | 15.00 | -58.00 | -26.00 | 9.81 | -58.86 | -21.48 | 526 | Anterior | Right | V |
|  | 2818 | 2.74 | -2.00 | -66.00 | -27.00 | 2.42 | -66.72 | -24.88 | 445 | Posterior | Vermis | VI |
|  | 2818 | 3.12 | 13.00 | -64.00 | -26.00 | 12.31 | -64.08 | -25.07 | 334 | Posterior | Right | VI |
|  | 2818 | 2.77 | 1.00 | -71.00 | -33.00 | 0.65 | -71.45 | -33.25 | 55 | Posterior | Vermis | CrusII |
|  | 2818 | 2.86 | 2.00 | -70.00 | -33.00 | 1.75 | -68.05 | -31.18 | 136 | Posterior | Vermis | VIIb |
|  | 2818 | 2.91 | 7.00 | -68.00 | -33.00 | 7.50 | -67.10 | -32.02 | 18 | Posterior | Right | VIIb |
|  | 2818 | 2.87 | 0.00 | -71.00 | -36.00 | 2.12 | -66.41 | -35.15 | 339 | Posterior | Vermis | VIIIa |
|  | 2818 | 2.95 | 8.00 | -65.00 | -32.00 | 8.16 | -64.50 | -32.63 | 6 | Posterior | Right | VIIIa |
|  | 2818 | 2.67 | 2.00 | -64.00 | -38.00 | 4.79 | -61.78 | -37.50 | 90 | Posterior | Vermis | VIIIb |
|  | 2818 | 2.89 | 8.00 | -61.00 | -37.00 | 8.00 | -61.00 | -37.00 | 1 | Posterior | Right | VIIIb |
|  | 2818 | 2.59 | -5.00 | -53.00 | -32.00 | -6.09 | -50.74 | -34.57 | 34 | Posterior | Left | IX |
|  | 2818 | 2.59 | 5.00 | -57.00 | -33.00 | 1.04 | -55.46 | -34.07 | 119 | Posterior | Vermis | IX |
|  | 2818 | 2.72 | 8.00 | -57.00 | -35.00 | 8.39 | -52.19 | -36.55 | 189 | Posterior | Right | IX |
|  | 2818 | 2.69 | -1.00 | -51.00 | -30.00 | 0.89 | -48.13 | -34.64 | 71 | Posterior | Vermis | X |
|  | 2818 | 2.88 | 9.00 | -66.00 | -31.00 | 12.57 | -61.72 | -29.84 | 184 | DN | Right | Dentate |
|  | 2818 | 2.93 | 10.00 | -58.00 | -25.00 | 8.08 | -57.93 | -29.40 | 154 | DN | Right | Interposed |
|  | 2818 | 2.98 | -3.00 | -52.00 | -28.00 | -3.00 | -52.00 | -28.00 | 1 | DN | Left | Fastigial |
|  | 2818 | 2.75 | 4.00 | -53.00 | -29.00 | 4.34 | -52.99 | -28.66 | 3 | DN | Right | Fastigial |
| Both hands without any covariate | 1515 | 2.93 | 0.00 | -51.00 | -28.00 | -1.52 | -49.01 | -27.34 | 15 | Anterior | Left | I_IV |
|  | 1515 | 2.68 | 9.00 | -48.00 | -21.00 | 7.29 | -50.56 | -20.15 | 53 | Anterior | Right | I_IV |
|  | 1515 | 3.07 | 15.00 | -58.00 | -26.00 | 10.65 | -58.62 | -21.55 | 405 | Anterior | Right | V |
|  | 1515 | 2.62 | -2.00 | -66.00 | -27.00 | 2.15 | -65.26 | -25.30 | 118 | Posterior | Vermis | VI |
|  | 1515 | 3.11 | 13.00 | -64.00 | -26.00 | 11.67 | -64.13 | -24.55 | 179 | Posterior | Right | VI |
|  | 1515 | 2.68 | 1.00 | -71.00 | -33.00 | 0.65 | -71.40 | -33.71 | 26 | Posterior | Vermis | CrusII |
|  | 1515 | 2.70 | 2.00 | -70.00 | -33.00 | 1.46 | -68.26 | -31.17 | 73 | Posterior | Vermis | VIIb |
|  | 1515 | 2.59 | 7.00 | -68.00 | -33.00 | 7.74 | -66.88 | -32.11 | 8 | Posterior | Right | VIIb |
|  | 1515 | 2.72 | 0.00 | -71.00 | -36.00 | 1.79 | -66.99 | -35.64 | 221 | Posterior | Vermis | VIIIa |
|  | 1515 | 2.61 | 8.00 | -65.00 | -32.00 | 8.31 | -64.97 | -32.31 | 3 | Posterior | Right | VIIIa |
|  | 1515 | 2.49 | 1.00 | -64.00 | -38.00 | 3.12 | -63.33 | -38.45 | 15 | Posterior | Vermis | VIIIb |
|  | 1515 | 2.58 | -5.00 | -53.00 | -32.00 | -6.08 | -50.52 | -35.10 | 27 | Posterior | Left | IX |
|  | 1515 | 2.52 | 5.00 | -57.00 | -33.00 | 0.70 | -55.08 | -34.54 | 86 | Posterior | Vermis | IX |
|  | 1515 | 2.57 | 8.00 | -57.00 | -35.00 | 7.68 | -50.92 | -37.07 | 127 | Posterior | Right | IX |
|  | 1515 | 2.60 | -1.00 | -51.00 | -30.00 | 1.08 | -48.07 | -34.75 | 67 | Posterior | Vermis | X |
|  | 1515 | 2.74 | 9.00 | -66.00 | -31.00 | 11.31 | -63.90 | -29.53 | 66 | DN | Right | Dentate |
|  | 1515 | 2.59 | 10.00 | -58.00 | -25.00 | 8.68 | -58.76 | -29.97 | 23 | DN | Right | Interposed |
|  | 1515 | 2.81 | -3.00 | -52.00 | -28.00 | -3.00 | -52.00 | -28.00 | 1 | DN | Left | Fastigial |
|  | 1515 | 2.47 | 4.00 | -53.00 | -29.00 | 4.50 | -52.50 | -28.50 | 2 | DN | Right | Fastigial |
| Assembly with all covariates | 2962 | 3.01 | 9.00 | -60.00 | -24.00 | 10.27 | -59.83 | -22.20 | 253 | Anterior | Right | V |
|  | 2962 | 2.90 | 3.00 | -64.00 | -29.00 | 2.07 | -65.13 | -27.06 | 155 | Posterior | Vermis | VI |
|  | 2962 | 2.89 | 12.00 | -63.00 | -25.00 | 12.03 | -63.38 | -24.87 | 123 | Posterior | Right | VI |
|  | 2962 | 2.74 | 3.00 | -70.00 | -34.00 | 1.78 | -71.17 | -35.19 | 12 | Posterior | Vermis | CrusII |
|  | 2962 | 2.83 | 4.00 | -73.00 | -39.00 | 4.00 | -73.00 | -39.00 | 1 | Posterior | Right | CrusII |
|  | 2962 | 2.90 | 2.00 | -70.00 | -33.00 | 1.68 | -67.75 | -31.08 | 103 | Posterior | Vermis | VIIb |
|  | 2962 | 2.90 | 8.00 | -67.00 | -33.00 | 8.36 | -67.56 | -34.54 | 23 | Posterior | Right | VIIb |
|  | 2962 | 2.96 | 1.00 | -63.00 | -36.00 | 2.46 | -66.15 | -35.64 | 502 | Posterior | Vermis | VIIIa |
|  | 2962 | 2.97 | 8.00 | -65.00 | -33.00 | 8.72 | -65.57 | -34.80 | 20 | Posterior | Right | VIIIa |
|  | 2962 | 2.82 | 1.00 | -63.00 | -37.00 | 2.86 | -62.56 | -39.19 | 270 | Posterior | Vermis | VIIIb |
|  | 2962 | 2.72 | 8.00 | -63.00 | -44.00 | 8.66 | -62.77 | -42.45 | 9 | Posterior | Right | VIIIb |
|  | 2962 | 2.95 | -6.00 | -52.00 | -39.00 | -5.33 | -51.91 | -37.35 | 382 | Posterior | Left | IX |
|  | 2962 | 2.88 | -1.00 | -53.00 | -37.00 | 0.24 | -55.55 | -36.71 | 432 | Posterior | Vermis | IX |
|  | 2962 | 2.94 | 5.00 | -51.00 | -39.00 | 6.21 | -51.93 | -38.27 | 208 | Posterior | Right | IX |
|  | 2962 | 3.08 | 1.00 | -47.00 | -38.00 | 1.53 | -47.65 | -37.09 | 231 | Posterior | Vermis | X |
|  | 2962 | 2.95 | 9.00 | -65.00 | -31.00 | 10.90 | -63.83 | -30.82 | 150 | DN | Right | Dentate |
|  | 2962 | 2.56 | -5.00 | -59.00 | -34.00 | -4.62 | -57.48 | -32.48 | 5 | DN | Left | Interposed |
|  | 2962 | 2.65 | 7.00 | -62.00 | -31.00 | 8.41 | -58.93 | -29.46 | 82 | DN | Right | Interposed |
|  | 2962 | 2.44 | -3.00 | -52.00 | -28.00 | -3.00 | -52.00 | -28.00 | 1 | DN | Left | Fastigial |
| Assembly without age at surgery as covariate | 2861 | 2.87 | 9.00 | -60.00 | -24.00 | 10.44 | -59.93 | -22.52 | 193 | Anterior | Right | V |
|  | 2861 | 2.84 | 3.00 | -64.00 | -29.00 | 2.05 | -65.09 | -26.96 | 153 | Posterior | Vermis | VI |
|  | 2861 | 2.77 | 12.00 | -63.00 | -25.00 | 11.90 | -63.32 | -24.87 | 71 | Posterior | Right | VI |
|  | 2861 | 2.76 | 2.00 | -72.00 | -37.00 | 1.84 | -71.28 | -35.29 | 11 | Posterior | Vermis | CrusII |
|  | 2861 | 2.87 | 4.00 | -73.00 | -39.00 | 4.00 | -73.00 | -39.00 | 1 | Posterior | Right | CrusII |
|  | 2861 | 2.82 | 2.00 | -70.00 | -33.00 | 1.65 | -67.75 | -31.07 | 99 | Posterior | Vermis | VIIb |
|  | 2861 | 2.72 | 7.00 | -68.00 | -33.00 | 8.15 | -67.16 | -33.62 | 13 | Posterior | Right | VIIb |
|  | 2861 | 2.92 | 1.00 | -63.00 | -36.00 | 2.38 | -66.27 | -35.97 | 504 | Posterior | Vermis | VIIIa |
|  | 2861 | 2.73 | 8.00 | -65.00 | -32.00 | 8.61 | -65.42 | -34.56 | 17 | Posterior | Right | VIIIa |
|  | 2861 | 2.82 | 1.00 | -63.00 | -37.00 | 2.71 | -62.58 | -39.25 | 278 | Posterior | Vermis | VIIIb |
|  | 2861 | 2.68 | 8.00 | -63.00 | -44.00 | 8.65 | -62.79 | -42.48 | 9 | Posterior | Right | VIIIb |
|  | 2861 | 3.04 | -6.00 | -52.00 | -39.00 | -5.29 | -51.96 | -37.53 | 423 | Posterior | Left | IX |
|  | 2861 | 2.91 | -1.00 | -53.00 | -37.00 | 0.42 | -55.43 | -36.95 | 507 | Posterior | Vermis | IX |
|  | 2861 | 2.98 | 5.00 | -51.00 | -39.00 | 6.00 | -51.78 | -38.48 | 203 | Posterior | Right | IX |
|  | 2861 | 3.12 | 1.00 | -47.00 | -38.00 | 1.50 | -47.80 | -36.92 | 255 | Posterior | Vermis | X |
|  | 2861 | 2.78 | 9.00 | -64.00 | -31.00 | 10.20 | -63.75 | -31.05 | 86 | DN | Right | Dentate |
|  | 2861 | 2.62 | -5.00 | -59.00 | -34.00 | -4.59 | -57.75 | -32.34 | 7 | DN | Left | Interposed |
|  | 2861 | 2.70 | 7.00 | -62.00 | -31.00 | 8.65 | -59.86 | -28.26 | 30 | DN | Right | Interposed |
|  | 2861 | 2.60 | -3.00 | -52.00 | -28.00 | -3.00 | -52.00 | -28.00 | 1 | DN | Left | Fastigial |
| Assembly without deep nuclei as covariate |  |  |  |  |  |  |  |  |  |  |  |  |
| Assembly without gender as covariate | 3118 | 3.02 | 9.00 | -60.00 | -24.00 | 10.32 | -59.83 | -22.14 | 265 | Anterior | Right | V |
|  | 3118 | 2.93 | 3.00 | -64.00 | -29.00 | 2.07 | -65.12 | -27.04 | 156 | Posterior | Vermis | VI |
|  | 3118 | 2.79 | 12.00 | -63.00 | -25.00 | 13.36 | -63.12 | -25.24 | 172 | Posterior | Right | VI |
|  | 3118 | 2.67 | 3.00 | -70.00 | -34.00 | 1.78 | -71.15 | -34.94 | 13 | Posterior | Vermis | CrusII |
|  | 3118 | 2.74 | 4.00 | -73.00 | -39.00 | 4.00 | -73.00 | -39.00 | 1 | Posterior | Right | CrusII |
|  | 3118 | 2.87 | 2.00 | -70.00 | -33.00 | 1.66 | -67.94 | -31.11 | 117 | Posterior | Vermis | VIIb |
|  | 3118 | 2.91 | 8.00 | -67.00 | -33.00 | 8.36 | -67.54 | -34.49 | 23 | Posterior | Right | VIIb |
|  | 3118 | 2.98 | 1.00 | -63.00 | -36.00 | 2.42 | -66.10 | -35.55 | 499 | Posterior | Vermis | VIIIa |
|  | 3118 | 2.99 | 8.00 | -65.00 | -33.00 | 8.72 | -65.57 | -34.79 | 20 | Posterior | Right | VIIIa |
|  | 3118 | 2.83 | 1.00 | -63.00 | -37.00 | 2.77 | -62.54 | -39.12 | 269 | Posterior | Vermis | VIIIb |
|  | 3118 | 2.70 | 8.00 | -63.00 | -44.00 | 8.67 | -62.77 | -42.44 | 9 | Posterior | Right | VIIIb |
|  | 3118 | 2.98 | -6.00 | -52.00 | -39.00 | -5.34 | -51.94 | -37.37 | 390 | Posterior | Left | IX |
|  | 3118 | 2.87 | -1.00 | -53.00 | -37.00 | 0.47 | -55.34 | -36.69 | 481 | Posterior | Vermis | IX |
|  | 3118 | 2.95 | 5.00 | -51.00 | -39.00 | 6.16 | -51.88 | -38.26 | 210 | Posterior | Right | IX |
|  | 3118 | 3.05 | 1.00 | -47.00 | -38.00 | 1.53 | -47.80 | -36.92 | 254 | Posterior | Vermis | X |
|  | 3118 | 2.98 | 9.00 | -65.00 | -31.00 | 10.90 | -63.82 | -30.82 | 151 | DN | Right | Dentate |
|  | 3118 | 2.60 | -5.00 | -59.00 | -34.00 | -4.62 | -57.48 | -32.48 | 5 | DN | Left | Interposed |
|  | 3118 | 2.67 | 7.00 | -62.00 | -31.00 | 8.41 | -58.93 | -29.45 | 82 | DN | Right | Interposed |
|  | 3118 | 2.46 | -3.00 | -52.00 | -28.00 | -3.00 | -52.00 | -28.00 | 1 | DN | Left | Fastigial |
| Assembly without treatment as covariate | 2785 | 2.61 | 8.00 | -60.00 | -25.00 | 10.54 | -60.26 | -23.26 | 79 | Anterior | Right | V |
|  | 2785 | 2.78 | 3.00 | -64.00 | -29.00 | 1.72 | -65.37 | -27.15 | 138 | Posterior | Vermis | VI |
|  | 2785 | 2.51 | 13.00 | -62.00 | -26.00 | 11.93 | -63.12 | -24.95 | 52 | Posterior | Right | VI |
|  | 2785 | 2.76 | 2.00 | -72.00 | -37.00 | 1.87 | -71.28 | -35.32 | 11 | Posterior | Vermis | CrusII |
|  | 2785 | 2.91 | 4.00 | -73.00 | -39.00 | 4.00 | -73.00 | -39.00 | 1 | Posterior | Right | CrusII |
|  | 2785 | 2.88 | 2.00 | -70.00 | -33.00 | 1.61 | -67.78 | -31.04 | 98 | Posterior | Vermis | VIIb |
|  | 2785 | 2.70 | 9.00 | -69.00 | -42.00 | 8.33 | -67.60 | -34.62 | 18 | Posterior | Right | VIIb |
|  | 2785 | 2.96 | 1.00 | -63.00 | -36.00 | 2.51 | -66.19 | -35.89 | 504 | Posterior | Vermis | VIIIa |
|  | 2785 | 2.80 | 8.00 | -65.00 | -32.00 | 8.63 | -65.46 | -34.62 | 17 | Posterior | Right | VIIIa |
|  | 2785 | 2.87 | 2.00 | -64.00 | -38.00 | 2.89 | -62.58 | -39.24 | 277 | Posterior | Vermis | VIIIb |
|  | 2785 | 2.75 | 8.00 | -63.00 | -44.00 | 8.67 | -62.90 | -42.49 | 12 | Posterior | Right | VIIIb |
|  | 2785 | 3.00 | -6.00 | -52.00 | -39.00 | -5.31 | -52.04 | -37.64 | 427 | Posterior | Left | IX |
|  | 2785 | 2.92 | -1.00 | -53.00 | -37.00 | 0.45 | -55.41 | -36.92 | 503 | Posterior | Vermis | IX |
|  | 2785 | 3.01 | 5.00 | -51.00 | -39.00 | 6.20 | -51.95 | -38.40 | 224 | Posterior | Right | IX |
|  | 2785 | 3.13 | 1.00 | -47.00 | -38.00 | 1.51 | -47.79 | -36.92 | 256 | Posterior | Vermis | X |
|  | 2785 | 2.73 | 9.00 | -64.00 | -31.00 | 10.36 | -64.04 | -31.35 | 90 | DN | Right | Dentate |
|  | 2785 | 2.62 | -5.00 | -59.00 | -34.00 | -4.59 | -57.75 | -32.34 | 7 | DN | Left | Interposed |
|  | 2785 | 2.64 | 7.00 | -62.00 | -31.00 | 8.29 | -58.62 | -29.53 | 70 | DN | Right | Interposed |
|  | 2785 | 2.55 | -3.00 | -52.00 | -28.00 | -3.00 | -52.00 | -28.00 | 1 | DN | Left | Fastigial |
| Assembly without any covariate |  |  |  |  |  |  |  |  |  |  |  |  |
| Total score with all covariates | 1555 | 2.61 | 2.00 | -57.00 | -18.00 | 2.00 | -57.00 | -18.00 | 1 | Anterior | Right | I_IV |
|  | 1555 | 3.09 | 0.00 | -64.00 | -20.00 | 0.00 | -62.87 | -18.79 | 3 | Anterior | Left | V |
|  | 1555 | 3.04 | 5.00 | -61.00 | -18.00 | 5.50 | -61.17 | -21.51 | 154 | Anterior | Right | V |
|  | 1555 | 2.74 | -5.00 | -65.00 | -27.00 | -5.00 | -65.00 | -27.00 | 1 | Posterior | Left | VI |
|  | 1555 | 3.11 | -2.00 | -66.00 | -29.00 | 2.46 | -64.69 | -25.82 | 297 | Posterior | Vermis | VI |
|  | 1555 | 3.22 | 13.00 | -61.00 | -26.00 | 9.21 | -63.48 | -25.37 | 39 | Posterior | Right | VI |
|  | 1555 | 2.74 | -5.00 | -67.00 | -32.00 | -5.00 | -67.00 | -32.00 | 1 | Posterior | Left | VIIb |
|  | 1555 | 2.93 | -2.00 | -66.00 | -31.00 | 1.43 | -66.98 | -30.65 | 83 | Posterior | Vermis | VIIb |
|  | 1555 | 2.93 | 8.00 | -67.00 | -31.00 | 7.80 | -67.11 | -32.44 | 25 | Posterior | Right | VIIb |
|  | 1555 | 2.66 | -8.00 | -66.00 | -36.00 | -7.07 | -66.19 | -35.14 | 11 | Posterior | Left | VIIIa |
|  | 1555 | 2.92 | 0.00 | -67.00 | -36.00 | 1.47 | -65.44 | -35.13 | 383 | Posterior | Vermis | VIIIa |
|  | 1555 | 2.67 | 8.00 | -65.00 | -32.00 | 8.68 | -65.45 | -35.17 | 14 | Posterior | Right | VIIIa |
|  | 1555 | 2.72 | 5.00 | -65.00 | -41.00 | 1.88 | -63.59 | -40.47 | 117 | Posterior | Vermis | VIIIb |
|  | 1555 | 2.50 | 8.00 | -65.00 | -46.00 | 8.00 | -65.00 | -46.00 | 1 | Posterior | Right | VIIIb |
|  | 1555 | 2.57 | -3.00 | -60.00 | -45.00 | -3.67 | -59.02 | -44.12 | 9 | Posterior | Left | IX |
|  | 1555 | 2.56 | -2.00 | -60.00 | -45.00 | -1.00 | -60.13 | -43.73 | 27 | Posterior | Vermis | IX |
|  | 1555 | 2.67 | -14.00 | -64.00 | -32.00 | -13.44 | -60.44 | -33.59 | 113 | DN | Left | Dentate |
|  | 1555 | 2.75 | 14.00 | -62.00 | -28.00 | 13.71 | -61.30 | -30.25 | 251 | DN | Right | Dentate |
|  | 1555 | 2.72 | 10.00 | -59.00 | -26.00 | 8.73 | -60.96 | -29.41 | 25 | DN | Right | Interposed |
| Total score without age at surgery as covariate | 1747 | 2.47 | 0.00 | -55.00 | -15.00 | -1.06 | -56.18 | -16.25 | 15 | Anterior | Left | I_IV |
|  | 1747 | 2.45 | 2.00 | -57.00 | -18.00 | 2.42 | -56.67 | -16.42 | 12 | Posterior | Right | I |
|  | 1747 | 2.52 | 0.00 | -64.00 | -20.00 | -0.74 | -60.79 | -17.98 | 30 | Anterior | Left | V |
|  | 1747 | 2.99 | 6.00 | -61.00 | -19.00 | 5.13 | -61.03 | -21.05 | 189 | Anterior | Right | V |
|  | 1747 | 2.61 | -5.00 | -65.00 | -27.00 | -5.00 | -65.00 | -27.00 | 1 | Posterior | Left | VI |
|  | 1747 | 3.06 | -2.00 | -66.00 | -27.00 | 2.55 | -64.80 | -25.79 | 310 | Posterior | Vermis | VI |
|  | 1747 | 3.33 | 13.00 | -61.00 | -26.00 | 9.21 | -63.85 | -25.54 | 45 | Posterior | Right | VI |
|  | 1747 | 2.61 | -5.00 | -67.00 | -32.00 | -5.00 | -67.00 | -32.00 | 1 | Posterior | Left | VIIb |
|  | 1747 | 2.98 | 2.00 | -67.00 | -31.00 | 1.73 | -66.99 | -30.60 | 82 | Posterior | Vermis | VIIb |
|  | 1747 | 3.18 | 8.00 | -67.00 | -31.00 | 7.85 | -67.13 | -32.49 | 27 | Posterior | Right | VIIb |
|  | 1747 | 2.61 | -6.00 | -66.00 | -33.00 | -6.00 | -66.50 | -33.00 | 4 | Posterior | Left | VIIIa |
|  | 1747 | 2.94 | 0.00 | -67.00 | -36.00 | 2.02 | -65.39 | -34.95 | 359 | Posterior | Vermis | VIIIa |
|  | 1747 | 2.79 | 8.00 | -65.00 | -32.00 | 8.93 | -65.31 | -35.01 | 23 | Posterior | Right | VIIIa |
|  | 1747 | 2.73 | 1.00 | -64.00 | -38.00 | 2.28 | -63.42 | -39.83 | 85 | Posterior | Vermis | VIIIb |
|  | 1747 | 2.45 | 13.00 | -61.00 | -43.00 | 13.00 | -61.00 | -42.50 | 2 | Posterior | Right | VIIIb |
|  | 1747 | 2.46 | -5.00 | -61.00 | -42.00 | -5.00 | -61.00 | -42.00 | 1 | Posterior | Left | IX |
|  | 1747 | 2.58 | 0.00 | -62.00 | -45.00 | -0.47 | -61.53 | -44.05 | 2 | Posterior | Vermis | IX |
|  | 1747 | 2.67 | -7.00 | -66.00 | -33.00 | -7.00 | -66.00 | -32.02 | 3 | DN | Left | Dentate |
|  | 1747 | 2.84 | 14.00 | -62.00 | -28.00 | 15.00 | -60.41 | -32.07 | 528 | DN | Right | Dentate |
|  | 1747 | 2.79 | 10.00 | -59.00 | -26.00 | 8.93 | -60.56 | -29.08 | 28 | DN | Right | Interposed |
| Total score without deep nuclei as covariate |  |  |  |  |  |  |  |  |  |  |  |  |
| Total score without gender as covariate | 1729 | 2.49 | 2.00 | -57.00 | -18.00 | 2.00 | -57.00 | -18.00 | 1 | Anterior | Right | I_IV |
|  | 1729 | 2.57 | 0.00 | -64.00 | -20.00 | -0.30 | -62.38 | -19.14 | 16 | Anterior | Left | V |
|  | 1729 | 2.97 | 5.00 | -61.00 | -18.00 | 5.18 | -61.21 | -21.08 | 172 | Anterior | Right | V |
|  | 1729 | 2.68 | -5.00 | -65.00 | -27.00 | -5.00 | -65.00 | -27.00 | 1 | Posterior | Left | VI |
|  | 1729 | 3.06 | -2.00 | -66.00 | -29.00 | 2.51 | -64.88 | -25.78 | 309 | Posterior | Vermis | VI |
|  | 1729 | 3.22 | 8.00 | -67.00 | -26.00 | 9.09 | -63.80 | -25.44 | 43 | Posterior | Right | VI |
|  | 1729 | 2.63 | -1.00 | -73.00 | -34.00 | -1.26 | -72.37 | -32.75 | 26 | Posterior | Vermis | CrusII |
|  | 1729 | 2.68 | -5.00 | -67.00 | -32.00 | -5.00 | -67.00 | -32.00 | 1 | Posterior | Left | VIIb |
|  | 1729 | 2.92 | 6.00 | -68.00 | -31.00 | 1.84 | -67.18 | -30.79 | 82 | Posterior | Vermis | VIIb |
|  | 1729 | 2.99 | 8.00 | -67.00 | -31.00 | 7.80 | -67.14 | -32.29 | 25 | Posterior | Right | VIIb |
|  | 1729 | 2.69 | -8.00 | -66.00 | -36.00 | -7.15 | -66.09 | -35.46 | 13 | Posterior | Left | VIIIa |
|  | 1729 | 2.89 | 0.00 | -67.00 | -36.00 | 1.58 | -65.70 | -35.49 | 396 | Posterior | Vermis | VIIIa |
|  | 1729 | 2.73 | 8.00 | -65.00 | -32.00 | 8.61 | -65.44 | -34.93 | 14 | Posterior | Right | VIIIa |
|  | 1729 | 2.71 | 5.00 | -65.00 | -41.00 | 2.19 | -63.70 | -40.76 | 127 | Posterior | Vermis | VIIIb |
|  | 1729 | 2.55 | 8.00 | -65.00 | -46.00 | 7.68 | -64.35 | -46.00 | 3 | Posterior | Right | VIIIb |
|  | 1729 | 2.58 | -3.00 | -60.00 | -45.00 | -3.50 | -59.02 | -44.13 | 8 | Posterior | Left | IX |
|  | 1729 | 2.53 | 0.00 | -62.00 | -45.00 | -0.51 | -59.98 | -43.58 | 32 | Posterior | Vermis | IX |
|  | 1729 | 2.62 | -9.00 | -67.00 | -36.00 | -13.39 | -60.59 | -33.58 | 102 | DN | Left | Dentate |
|  | 1729 | 2.75 | 14.00 | -62.00 | -28.00 | 14.47 | -60.91 | -31.05 | 333 | DN | Right | Dentate |
|  | 1729 | 2.71 | 10.00 | -59.00 | -26.00 | 8.73 | -60.95 | -29.40 | 25 | DN | Right | Interposed |
| Total score without treatment as covariate | 1054 | 2.44 | 0.00 | -64.00 | -20.00 | 0.00 | -64.00 | -19.50 | 2 | Anterior | Left | V |
|  | 1054 | 2.54 | 5.00 | -61.00 | -25.00 | 4.15 | -61.80 | -21.80 | 43 | Anterior | Right | V |
|  | 1054 | 2.56 | -5.00 | -65.00 | -27.00 | -5.00 | -65.00 | -27.00 | 1 | Posterior | Left | VI |
|  | 1054 | 2.68 | -2.00 | -66.00 | -29.00 | 1.54 | -64.78 | -26.30 | 224 | Posterior | Vermis | VI |
|  | 1054 | 2.57 | 8.00 | -67.00 | -26.00 | 7.02 | -64.72 | -27.30 | 3 | Posterior | Right | VI |
|  | 1054 | 2.56 | -5.00 | -67.00 | -32.00 | -5.00 | -67.00 | -32.00 | 1 | Posterior | Left | VIIb |
|  | 1054 | 2.80 | 0.00 | -67.00 | -32.00 | 1.13 | -67.12 | -30.69 | 69 | Posterior | Vermis | VIIb |
|  | 1054 | 2.54 | 7.00 | -68.00 | -33.00 | 7.30 | -67.00 | -31.60 | 10 | Posterior | Right | VIIb |
|  | 1054 | 2.59 | -8.00 | -66.00 | -36.00 | -7.08 | -66.30 | -35.18 | 10 | Posterior | Left | VIIIa |
|  | 1054 | 2.87 | 0.00 | -67.00 | -36.00 | 1.38 | -65.47 | -35.47 | 364 | Posterior | Vermis | VIIIa |
|  | 1054 | 2.61 | 8.00 | -66.00 | -45.00 | 8.16 | -64.67 | -36.76 | 6 | Posterior | Right | VIIIa |
|  | 1054 | 2.72 | 5.00 | -65.00 | -41.00 | 2.59 | -63.66 | -40.99 | 154 | Posterior | Vermis | VIIIb |
|  | 1054 | 2.67 | 8.00 | -65.00 | -46.00 | 7.76 | -64.02 | -46.00 | 4 | Posterior | Right | VIIIb |
|  | 1054 | 2.57 | -3.00 | -60.00 | -45.00 | -3.94 | -58.59 | -43.72 | 23 | Posterior | Left | IX |
|  | 1054 | 2.62 | -2.00 | -60.00 | -45.00 | -0.03 | -59.92 | -43.82 | 54 | Posterior | Vermis | IX |
|  | 1054 | 2.64 | 10.00 | -59.00 | -45.00 | 8.18 | -60.11 | -44.50 | 27 | Posterior | Right | IX |
|  | 1054 | 2.66 | -9.00 | -67.00 | -36.00 | -8.10 | -66.00 | -34.43 | 10 | DN | Left | Dentate |
|  | 1054 | 2.58 | 18.00 | -55.00 | -31.00 | 15.36 | -57.26 | -30.28 | 41 | DN | Right | Dentate |
|  | 1054 | 2.48 | 9.00 | -59.00 | -26.00 | 8.99 | -60.26 | -29.01 | 8 | DN | Right | Interposed |
| Total score without any covariate |  |  |  |  |  |  |  |  |  |  |  |  |
| Kinetic function with all covariates | 1354 | 2.70 | 2.00 | -57.00 | -18.00 | 2.00 | -57.00 | -18.00 | 1 | Anterior | Right | I_IV |
|  | 1354 | 2.96 | 0.00 | -64.00 | -20.00 | 0.00 | -62.83 | -18.77 | 3 | Anterior | Left | V |
|  | 1354 | 2.82 | 5.00 | -61.00 | -18.00 | 5.45 | -61.24 | -21.32 | 144 | Anterior | Right | V |
|  | 1354 | 2.91 | 0.00 | -66.00 | -29.00 | 2.42 | -65.15 | -25.89 | 329 | Posterior | Vermis | VI |
|  | 1354 | 2.96 | 13.00 | -61.00 | -26.00 | 9.32 | -63.57 | -25.46 | 40 | Posterior | Right | VI |
|  | 1354 | 2.63 | 1.00 | -71.00 | -33.00 | -0.59 | -72.20 | -32.45 | 31 | Posterior | Vermis | CrusII |
|  | 1354 | 2.93 | 2.00 | -66.00 | -31.00 | 1.73 | -67.15 | -30.74 | 90 | Posterior | Vermis | VIIb |
|  | 1354 | 2.64 | 8.00 | -67.00 | -31.00 | 7.88 | -67.22 | -32.76 | 27 | Posterior | Right | VIIb |
|  | 1354 | 2.94 | 2.00 | -67.00 | -35.00 | 2.49 | -66.00 | -35.56 | 397 | Posterior | Vermis | VIIIa |
|  | 1354 | 2.64 | 8.00 | -65.00 | -32.00 | 8.43 | -65.29 | -34.24 | 9 | Posterior | Right | VIIIa |
|  | 1354 | 2.81 | 1.00 | -63.00 | -37.00 | 3.93 | -62.96 | -38.85 | 107 | Posterior | Vermis | VIIIb |
|  | 1354 | 2.49 | 4.00 | -61.00 | -42.00 | 4.00 | -61.00 | -42.00 | 1 | Posterior | Vermis | IX |
|  | 1354 | 2.50 | 7.00 | -61.00 | -41.00 | 7.00 | -61.00 | -41.00 | 1 | Posterior | Right | IX |
|  | 1354 | 2.73 | 14.00 | -62.00 | -28.00 | 12.09 | -62.76 | -29.73 | 155 | DN | Right | Dentate |
|  | 1354 | 2.61 | 10.00 | -59.00 | -26.00 | 8.81 | -60.91 | -29.19 | 19 | DN | Right | Interposed |
| Kinetic function without age at surgery as covariate | 1536 | 2.56 | 2.00 | -57.00 | -18.00 | 2.00 | -57.00 | -18.00 | 1 | Anterior | Right | I_IV |
|  | 1536 | 3.05 | 0.00 | -64.00 | -20.00 | 0.00 | -62.87 | -18.79 | 3 | Anterior | Left | V |
|  | 1536 | 2.88 | 5.00 | -61.00 | -18.00 | 5.82 | -61.17 | -21.46 | 148 | Anterior | Right | V |
|  | 1536 | 2.90 | 0.00 | -66.00 | -29.00 | 2.64 | -65.40 | -25.85 | 325 | Posterior | Vermis | VI |
|  | 1536 | 3.09 | 13.00 | -61.00 | -26.00 | 9.51 | -63.71 | -25.52 | 50 | Posterior | Right | VI |
|  | 1536 | 2.67 | 0.00 | -72.00 | -31.00 | 0.01 | -72.36 | -32.03 | 45 | Posterior | Vermis | CrusII |
|  | 1536 | 2.99 | 2.00 | -67.00 | -31.00 | 2.29 | -67.14 | -30.66 | 88 | Posterior | Vermis | VIIb |
|  | 1536 | 2.95 | 8.00 | -67.00 | -31.00 | 7.92 | -67.21 | -32.82 | 28 | Posterior | Right | VIIb |
|  | 1536 | 2.99 | 2.00 | -67.00 | -35.00 | 3.05 | -65.62 | -35.30 | 351 | Posterior | Vermis | VIIIa |
|  | 1536 | 2.73 | 8.00 | -65.00 | -32.00 | 8.91 | -65.64 | -35.16 | 18 | Posterior | Right | VIIIa |
|  | 1536 | 2.85 | 4.00 | -63.00 | -41.00 | 4.37 | -62.57 | -38.51 | 105 | Posterior | Vermis | VIIIb |
|  | 1536 | 2.52 | 5.00 | -60.00 | -39.00 | 5.34 | -59.34 | -37.66 | 3 | Posterior | Vermis | IX |
|  | 1536 | 2.49 | 7.00 | -61.00 | -41.00 | 7.00 | -61.00 | -41.00 | 1 | Posterior | Right | IX |
|  | 1536 | 2.85 | 14.00 | -62.00 | -28.00 | 14.25 | -61.82 | -31.10 | 349 | DN | Right | Dentate |
|  | 1536 | 2.70 | 10.00 | -58.00 | -25.00 | 8.84 | -60.94 | -29.50 | 21 | DN | Right | Interposed |
| Kinetic function without deep nuclei as covariate |  |  |  |  |  |  |  |  |  |  |  |  |
| Kinetic function without gender as covariate | 1457 | 2.60 | 2.00 | -57.00 | -18.00 | 2.00 | -57.00 | -18.00 | 1 | Anterior | Right | I_IV |
|  | 1457 | 3.00 | 0.00 | -64.00 | -20.00 | 0.00 | -62.86 | -18.79 | 3 | Anterior | Left | V |
|  | 1457 | 2.82 | 5.00 | -61.00 | -18.00 | 5.59 | -61.19 | -21.39 | 146 | Anterior | Right | V |
|  | 1457 | 2.87 | 0.00 | -66.00 | -29.00 | 2.44 | -65.49 | -25.81 | 367 | Posterior | Vermis | VI |
|  | 1457 | 2.99 | 13.00 | -61.00 | -26.00 | 9.21 | -63.82 | -25.49 | 43 | Posterior | Right | VI |
|  | 1457 | 2.71 | 1.00 | -71.00 | -34.00 | 0.02 | -72.30 | -32.35 | 46 | Posterior | Vermis | CrusII |
|  | 1457 | 2.94 | 2.00 | -67.00 | -31.00 | 2.19 | -67.22 | -30.73 | 92 | Posterior | Vermis | VIIb |
|  | 1457 | 2.71 | 8.00 | -67.00 | -31.00 | 7.87 | -67.23 | -32.68 | 26 | Posterior | Right | VIIb |
|  | 1457 | 2.95 | 2.00 | -67.00 | -35.00 | 2.73 | -66.13 | -35.78 | 394 | Posterior | Vermis | VIIIa |
|  | 1457 | 2.69 | 8.00 | -65.00 | -32.00 | 8.32 | -65.18 | -33.95 | 9 | Posterior | Right | VIIIa |
|  | 1457 | 2.81 | 2.00 | -64.00 | -38.00 | 4.24 | -63.00 | -39.09 | 106 | Posterior | Vermis | VIIIb |
|  | 1457 | 2.52 | 4.00 | -61.00 | -42.00 | 4.00 | -61.00 | -42.00 | 1 | Posterior | Vermis | IX |
|  | 1457 | 2.60 | 7.00 | -61.00 | -41.00 | 7.00 | -61.00 | -41.00 | 1 | Posterior | Right | IX |
|  | 1457 | 2.71 | 14.00 | -62.00 | -28.00 | 13.36 | -62.09 | -30.17 | 200 | DN | Right | Dentate |
|  | 1457 | 2.57 | 10.00 | -58.00 | -25.00 | 8.71 | -60.91 | -29.02 | 22 | DN | Right | Interposed |
| Kinetic function without treatment as covariate |  |  |  |  |  |  |  |  |  |  |  |  |
| Kinetic function without any covariate |  |  |  |  |  |  |  |  |  |  |  |  |
| OM disorders with all covariates |  |  |  |  |  |  |  |  |  |  |  |  |
| OM disorders without age at surgery as covariate |  |  |  |  |  |  |  |  |  |  |  |  |
| OM disorders without deep nuclei as covariate |  |  |  |  |  |  |  |  |  |  |  |  |
| OM diorders without gender as covatiate |  |  |  |  |  |  |  |  |  |  |  |  |
| OM disorders without treatment as covariate |  |  |  |  |  |  |  |  |  |  |  |  |
| OM disorders without any covariate |  |  |  |  |  |  |  |  |  |  |  |  |
| Posture and gait with all covariates | 2833 | 2.61 | -3.00 | -56.00 | -18.00 | -2.53 | -54.65 | -16.69 | 80 | Anterior | Left | I_IV |
|  | 2833 | 2.64 | 1.00 | -52.00 | -16.00 | 2.32 | -56.41 | -17.08 | 19 | Anterior | Right | I_IV |
|  | 2833 | 2.73 | 0.00 | -64.00 | -20.00 | -2.45 | -60.60 | -19.86 | 188 | Anterior | Left | V |
|  | 2833 | 3.03 | 8.00 | -61.00 | -25.00 | 4.72 | -60.71 | -20.93 | 270 | Anterior | Right | V |
|  | 2833 | 2.79 | -5.00 | -65.00 | -27.00 | -6.90 | -64.05 | -25.06 | 56 | Posterior | Left | VI |
|  | 2833 | 3.02 | -2.00 | -66.00 | -29.00 | 1.24 | -64.87 | -25.20 | 503 | Posterior | Vermis | VI |
|  | 2833 | 3.23 | 7.00 | -66.00 | -30.00 | 9.11 | -63.95 | -25.52 | 52 | Posterior | Right | VI |
|  | 2833 | 3.81 | -5.00 | -67.00 | -32.00 | -5.00 | -67.00 | -32.00 | 1 | Posterior | Left | VIIb |
|  | 2833 | 3.10 | -2.00 | -66.00 | -31.00 | 0.98 | -67.12 | -30.74 | 81 | Posterior | Vermis | VIIb |
|  | 2833 | 3.32 | 7.00 | -68.00 | -32.00 | 7.90 | -67.16 | -32.56 | 28 | Posterior | Right | VIIb |
|  | 2833 | 3.25 | -6.00 | -66.00 | -33.00 | -7.02 | -66.19 | -35.05 | 19 | Posterior | Left | VIIIa |
|  | 2833 | 3.00 | -5.00 | -65.00 | -36.00 | 0.16 | -65.40 | -34.97 | 378 | Posterior | Vermis | VIIIa |
|  | 2833 | 2.90 | 9.00 | -66.00 | -37.00 | 8.92 | -65.39 | -35.51 | 25 | Posterior | Right | VIIIa |
|  | 2833 | 2.62 | -6.00 | -63.00 | -44.00 | -7.89 | -62.17 | -41.60 | 13 | Posterior | Left | VIIIb |
|  | 2833 | 2.85 | 2.00 | -65.00 | -41.00 | -0.23 | -63.64 | -42.01 | 193 | Posterior | Vermis | VIIIb |
|  | 2833 | 2.68 | 8.00 | -65.00 | -46.00 | 7.53 | -64.53 | -46.00 | 2 | Posterior | Right | VIIIb |
|  | 2833 | 2.81 | -3.00 | -60.00 | -45.00 | -5.02 | -58.19 | -43.58 | 113 | Posterior | Left | IX |
|  | 2833 | 2.88 | -2.00 | -60.00 | -45.00 | -0.02 | -59.80 | -43.77 | 102 | Posterior | Vermis | IX |
|  | 2833 | 2.86 | 3.00 | -56.00 | -47.00 | 3.33 | -59.04 | -46.78 | 62 | Posterior | Right | IX |
|  | 2833 | 2.84 | -7.00 | -66.00 | -32.00 | -11.61 | -61.89 | -32.73 | 315 | DN | Left | Dentate |
|  | 2833 | 2.83 | 9.00 | -65.00 | -29.00 | 13.83 | -61.67 | -30.85 | 311 | DN | Right | Dentate |
|  | 2833 | 2.78 | 9.00 | -59.00 | -26.00 | 8.65 | -60.97 | -29.03 | 22 | DN | Right | Interposed |
| Posture and gait without age at surgery as covariate | 3067 | 2.67 | 0.00 | -53.00 | -16.00 | -2.44 | -54.53 | -16.69 | 83 | Anterior | Left | I_IV |
|  | 3067 | 2.72 | 1.00 | -52.00 | -16.00 | 4.19 | -53.76 | -18.77 | 22 | Anterior | Right | I_IV |
|  | 3067 | 2.74 | 0.00 | -64.00 | -20.00 | -2.52 | -60.54 | -19.83 | 189 | Anterior | Left | V |
|  | 3067 | 3.06 | 6.00 | -61.00 | -19.00 | 4.97 | -60.68 | -21.03 | 280 | Anterior | Right | V |
|  | 3067 | 2.75 | -5.00 | -65.00 | -27.00 | -6.85 | -64.11 | -25.01 | 54 | Posterior | Left | VI |
|  | 3067 | 2.99 | -2.00 | -66.00 | -29.00 | 1.30 | -64.89 | -25.22 | 507 | Posterior | Vermis | VI |
|  | 3067 | 3.35 | 7.00 | -66.00 | -30.00 | 9.33 | -63.87 | -25.59 | 56 | Posterior | Right | VI |
|  | 3067 | 3.70 | -5.00 | -67.00 | -32.00 | -5.00 | -67.00 | -32.00 | 1 | Posterior | Left | VIIb |
|  | 3067 | 3.05 | 7.00 | -67.00 | -30.00 | 1.26 | -67.02 | -30.70 | 94 | Posterior | Vermis | VIIb |
|  | 3067 | 3.45 | 7.00 | -68.00 | -32.00 | 7.98 | -67.22 | -32.77 | 31 | Posterior | Right | VIIb |
|  | 3067 | 3.12 | -6.00 | -66.00 | -33.00 | -6.93 | -66.21 | -34.90 | 18 | Posterior | Left | VIIIa |
|  | 3067 | 2.97 | 0.00 | -67.00 | -36.00 | 0.40 | -65.46 | -34.78 | 377 | Posterior | Vermis | VIIIa |
|  | 3067 | 2.91 | 9.00 | -66.00 | -37.00 | 9.14 | -65.22 | -35.65 | 36 | Posterior | Right | VIIIa |
|  | 3067 | 2.59 | -6.00 | -63.00 | -44.00 | -6.00 | -63.00 | -44.00 | 1 | Posterior | Left | VIIIb |
|  | 3067 | 2.83 | 0.00 | -62.00 | -44.00 | -0.26 | -63.52 | -41.42 | 128 | Posterior | Vermis | VIIIb |
|  | 3067 | 2.52 | 12.00 | -63.00 | -41.00 | 12.66 | -61.68 | -41.99 | 3 | Posterior | Right | VIIIb |
|  | 3067 | 2.71 | -3.00 | -60.00 | -45.00 | -4.93 | -58.23 | -43.52 | 82 | Posterior | Left | IX |
|  | 3067 | 2.80 | 0.00 | -62.00 | -45.00 | -0.54 | -59.88 | -43.83 | 69 | Posterior | Vermis | IX |
|  | 3067 | 2.66 | 3.00 | -56.00 | -47.00 | 6.52 | -53.65 | -46.38 | 133 | Posterior | Right | IX |
|  | 3067 | 2.77 | -7.00 | -66.00 | -32.00 | -11.74 | -61.53 | -32.58 | 276 | DN | Left | Dentate |
|  | 3067 | 2.81 | 9.00 | -65.00 | -29.00 | 15.31 | -60.00 | -32.59 | 602 | DN | Right | Dentate |
|  | 3067 | 2.84 | 9.00 | -59.00 | -26.00 | 8.66 | -60.93 | -29.25 | 25 | DN | Right | Interposed |
| Posture and gait without deep nuclei as covariate |  |  |  |  |  |  |  |  |  |  |  |  |
| Posture and gait without gender as covariate | 2967 | 2.65 | -3.00 | -56.00 | -18.00 | -2.48 | -54.66 | -16.70 | 82 | Anterior | Left | I_IV |
|  | 2967 | 2.56 | 1.00 | -52.00 | -16.00 | 2.56 | -55.34 | -16.65 | 37 | Anterior | Right | I_IV |
|  | 2967 | 2.75 | 0.00 | -64.00 | -20.00 | -2.47 | -60.61 | -19.83 | 188 | Anterior | Left | V |
|  | 2967 | 3.04 | 6.00 | -61.00 | -19.00 | 4.77 | -60.71 | -20.94 | 267 | Anterior | Right | V |
|  | 2967 | 2.81 | -5.00 | -65.00 | -27.00 | -6.90 | -64.06 | -25.04 | 56 | Posterior | Left | VI |
|  | 2967 | 3.01 | -2.00 | -66.00 | -29.00 | 1.26 | -64.94 | -25.18 | 502 | Posterior | Vermis | VI |
|  | 2967 | 3.28 | 7.00 | -66.00 | -30.00 | 9.16 | -63.97 | -25.56 | 54 | Posterior | Right | VI |
|  | 2967 | 3.76 | -5.00 | -67.00 | -32.00 | -5.00 | -67.00 | -32.00 | 1 | Posterior | Left | VIIb |
|  | 2967 | 3.07 | 7.00 | -67.00 | -30.00 | 1.20 | -67.18 | -30.77 | 84 | Posterior | Vermis | VIIb |
|  | 2967 | 3.38 | 7.00 | -68.00 | -32.00 | 7.89 | -67.16 | -32.55 | 28 | Posterior | Right | VIIb |
|  | 2967 | 3.26 | -8.00 | -66.00 | -36.00 | -7.03 | -66.18 | -35.19 | 20 | Posterior | Left | VIIIa |
|  | 2967 | 2.98 | -5.00 | -65.00 | -36.00 | 0.09 | -65.54 | -35.19 | 374 | Posterior | Vermis | VIIIa |
|  | 2967 | 2.96 | 9.00 | -66.00 | -37.00 | 8.92 | -65.40 | -35.46 | 24 | Posterior | Right | VIIIa |
|  | 2967 | 2.62 | -6.00 | -63.00 | -44.00 | -7.90 | -62.22 | -41.70 | 14 | Posterior | Left | VIIIb |
|  | 2967 | 2.85 | 2.00 | -65.00 | -41.00 | -0.10 | -63.65 | -42.06 | 199 | Posterior | Vermis | VIIIb |
|  | 2967 | 2.80 | 8.00 | -65.00 | -46.00 | 7.53 | -64.53 | -46.00 | 2 | Posterior | Right | VIIIb |
|  | 2967 | 2.84 | -3.00 | -60.00 | -45.00 | -4.83 | -58.46 | -43.62 | 102 | Posterior | Left | IX |
|  | 2967 | 2.92 | -2.00 | -60.00 | -45.00 | 0.19 | -59.80 | -43.87 | 100 | Posterior | Vermis | IX |
|  | 2967 | 2.82 | 3.00 | -56.00 | -47.00 | 3.98 | -58.73 | -46.49 | 96 | Posterior | Right | IX |
|  | 2967 | 2.85 | -8.00 | -65.00 | -35.00 | -11.39 | -62.16 | -32.57 | 289 | DN | Left | Dentate |
|  | 2967 | 2.79 | 9.00 | -65.00 | -29.00 | 14.84 | -60.63 | -31.71 | 424 | DN | Right | Dentate |
|  | 2967 | 2.76 | 8.00 | -63.00 | -30.00 | 8.68 | -61.01 | -29.32 | 24 | DN | Right | Interposed |
| Posture and gait without treatment as covariate | 1840 | 2.53 | -4.00 | -62.00 | -23.00 | -3.01 | -61.33 | -23.12 | 18 | Anterior | Left | V |
|  | 1840 | 2.65 | 7.00 | -61.00 | -25.00 | 5.56 | -61.64 | -21.96 | 112 | Anterior | Right | V |
|  | 1840 | 2.66 | -5.00 | -65.00 | -27.00 | -5.44 | -65.02 | -25.65 | 28 | Posterior | Left | VI |
|  | 1840 | 2.76 | -2.00 | -66.00 | -29.00 | 1.36 | -64.68 | -25.62 | 258 | Posterior | Vermis | VI |
|  | 1840 | 2.72 | 7.00 | -66.00 | -30.00 | 7.66 | -63.42 | -25.31 | 15 | Posterior | Right | VI |
|  | 1840 | 3.54 | -5.00 | -67.00 | -32.00 | -5.00 | -67.00 | -32.00 | 1 | Posterior | Left | VIIb |
|  | 1840 | 2.92 | -2.00 | -66.00 | -31.00 | 0.63 | -67.13 | -30.76 | 76 | Posterior | Vermis | VIIb |
|  | 1840 | 2.79 | 7.00 | -68.00 | -32.00 | 7.78 | -67.20 | -32.21 | 18 | Posterior | Right | VIIb |
|  | 1840 | 3.12 | -8.00 | -66.00 | -36.00 | -7.02 | -66.19 | -35.07 | 19 | Posterior | Left | VIIIa |
|  | 1840 | 2.90 | -5.00 | -65.00 | -36.00 | 0.12 | -65.47 | -35.42 | 375 | Posterior | Vermis | VIIIa |
|  | 1840 | 2.64 | 8.00 | -66.00 | -45.00 | 8.85 | -65.34 | -35.74 | 15 | Posterior | Right | VIIIa |
|  | 1840 | 2.76 | -6.00 | -63.00 | -44.00 | -7.15 | -62.61 | -43.82 | 5 | Posterior | Left | VIIIb |
|  | 1840 | 2.90 | 3.00 | -63.00 | -43.00 | 0.37 | -63.72 | -42.25 | 224 | Posterior | Vermis | VIIIb |
|  | 1840 | 2.89 | 8.00 | -65.00 | -46.00 | 7.67 | -64.37 | -46.00 | 3 | Posterior | Right | VIIIb |
|  | 1840 | 2.80 | -3.00 | -60.00 | -45.00 | -4.45 | -57.10 | -44.01 | 195 | Posterior | Left | IX |
|  | 1840 | 3.00 | -2.00 | -60.00 | -45.00 | 0.21 | -59.70 | -43.84 | 121 | Posterior | Vermis | IX |
|  | 1840 | 2.90 | 3.00 | -56.00 | -47.00 | 4.24 | -58.55 | -46.31 | 134 | Posterior | Right | IX |
|  | 1840 | 2.84 | -8.00 | -67.00 | -35.00 | -10.77 | -62.94 | -33.20 | 179 | DN | Left | Dentate |
|  | 1840 | 2.61 | 9.00 | -65.00 | -29.00 | 10.16 | -64.66 | -30.88 | 36 | DN | Right | Dentate |
|  | 1840 | 2.57 | 7.00 | -63.00 | -30.00 | 7.87 | -62.11 | -29.95 | 8 | DN | Right | Interposed |
| Posture and gait without any covariate | 894 | 2.53 | 12.00 | -42.00 | -26.00 | 10.90 | -41.20 | -25.39 | 30 | Anterior | Right | I_IV |
|  | 894 | 2.87 | 17.00 | -56.00 | -27.00 | 17.00 | -56.00 | -27.00 | 1 | Anterior | Right | V |
|  | 894 | 2.50 | 10.00 | -68.00 | -36.00 | 10.00 | -68.00 | -36.00 | 1 | Posterior | Right | VIIb |
|  | 894 | 2.58 | 6.00 | -67.00 | -38.00 | 4.20 | -66.73 | -40.02 | 39 | Posterior | Vermis | VIIIa |
|  | 894 | 2.63 | 9.00 | -66.00 | -37.00 | 9.70 | -66.30 | -36.96 | 7 | Posterior | Right | VIIIa |
|  | 894 | 2.47 | -6.00 | -63.00 | -44.00 | -6.00 | -63.00 | -44.00 | 1 | Posterior | Left | VIIIb |
|  | 894 | 2.76 | 0.00 | -62.00 | -44.00 | 0.32 | -63.75 | -42.18 | 130 | Posterior | Vermis | VIIIb |
|  | 894 | 2.60 | 8.00 | -65.00 | -46.00 | 8.00 | -65.00 | -46.00 | 1 | Posterior | Right | VIIIb |
|  | 894 | 2.64 | -3.00 | -60.00 | -45.00 | -4.24 | -56.81 | -44.11 | 128 | Posterior | Left | IX |
|  | 894 | 2.85 | 0.00 | -62.00 | -45.00 | -0.31 | -59.94 | -43.95 | 78 | Posterior | Vermis | IX |
|  | 894 | 2.72 | 3.00 | -56.00 | -47.00 | 7.40 | -52.47 | -46.16 | 262 | Posterior | Right | IX |
|  | 894 | 2.61 | 18.00 | -55.00 | -31.00 | 18.31 | -56.85 | -34.64 | 216 | DN | Right | Dentate |
| Speech with all covariates | 1410 | 2.73 | 12.00 | -41.00 | -28.00 | 11.12 | -40.38 | -26.77 | 20 | Anterior | Right | I_IV |
|  | 1410 | 3.65 | 7.00 | -67.00 | -45.00 | 7.47 | -66.53 | -44.53 | 2 | Posterior | Vermis | VIIIa |
|  | 1410 | 3.23 | 8.00 | -66.00 | -45.00 | 8.99 | -65.50 | -45.26 | 4 | Posterior | Right | VIIIa |
|  | 1410 | 3.01 | 7.00 | -65.00 | -46.00 | 6.00 | -64.72 | -44.79 | 21 | Posterior | Vermis | VIIIb |
|  | 1410 | 3.10 | 8.00 | -65.00 | -46.00 | 12.41 | -61.41 | -44.09 | 76 | Posterior | Right | VIIIb |
|  | 1410 | 2.89 | -8.00 | -47.00 | -49.00 | -7.42 | -48.69 | -45.65 | 346 | Posterior | Left | IX |
|  | 1410 | 2.67 | 0.00 | -59.00 | -46.00 | 0.46 | -60.62 | -45.75 | 12 | Posterior | Vermis | IX |
|  | 1410 | 3.04 | 10.00 | -58.00 | -45.00 | 7.88 | -52.09 | -46.35 | 370 | Posterior | Right | IX |
|  | 1410 | 2.48 | -12.00 | -51.00 | -39.00 | -12.45 | -52.44 | -37.87 | 22 | DN | Left | Dentate |
|  | 1410 | 3.04 | 17.00 | -56.00 | -41.00 | 17.30 | -57.28 | -35.04 | 533 | DN | Right | Dentate |
|  | 1410 | 2.70 | 11.00 | -58.00 | -26.00 | 10.76 | -57.28 | -26.00 | 4 | DN | Right | Interposed |
| Speech without age at surgery as covariate | 1385 | 2.80 | 12.00 | -41.00 | -28.00 | 11.12 | -40.38 | -26.76 | 20 | Anterior | Right | I_IV |
|  | 1385 | 3.57 | 7.00 | -67.00 | -45.00 | 7.47 | -66.53 | -44.53 | 2 | Posterior | Vermis | VIIIa |
|  | 1385 | 3.22 | 10.00 | -65.00 | -46.00 | 9.01 | -65.49 | -45.27 | 4 | Posterior | Right | VIIIa |
|  | 1385 | 3.24 | 7.00 | -65.00 | -46.00 | 6.44 | -65.05 | -44.91 | 12 | Posterior | Vermis | VIIIb |
|  | 1385 | 3.16 | 11.00 | -62.00 | -46.00 | 12.43 | -61.39 | -44.08 | 76 | Posterior | Right | VIIIb |
|  | 1385 | 2.92 | -8.00 | -47.00 | -49.00 | -7.47 | -48.58 | -45.62 | 341 | Posterior | Left | IX |
|  | 1385 | 2.49 | 2.00 | -61.00 | -46.00 | 2.00 | -61.00 | -46.00 | 1 | Posterior | Vermis | IX |
|  | 1385 | 3.10 | 10.00 | -58.00 | -45.00 | 8.23 | -51.71 | -46.24 | 369 | Posterior | Right | IX |
|  | 1385 | 2.51 | -13.00 | -51.00 | -40.00 | -12.45 | -52.43 | -37.88 | 22 | DN | Left | Dentate |
|  | 1385 | 3.07 | 17.00 | -56.00 | -41.00 | 17.29 | -57.26 | -35.06 | 534 | DN | Right | Dentate |
|  | 1385 | 2.78 | 11.00 | -58.00 | -26.00 | 10.76 | -57.28 | -26.00 | 4 | DN | Right | Interposed |
| Speech without deep nuclei as covariate | 1755 | 2.70 | 13.00 | -41.00 | -29.00 | 11.29 | -42.03 | -26.94 | 37 | Anterior | Right | I_IV |
|  | 1755 | 2.99 | 7.00 | -67.00 | -45.00 | 7.34 | -66.94 | -43.94 | 6 | Posterior | Vermis | VIIIa |
|  | 1755 | 2.99 | 8.00 | -66.00 | -45.00 | 9.69 | -65.68 | -44.47 | 7 | Posterior | Right | VIIIa |
|  | 1755 | 2.95 | 7.00 | -65.00 | -46.00 | 6.18 | -64.15 | -43.97 | 33 | Posterior | Vermis | VIIIb |
|  | 1755 | 3.08 | 8.00 | -65.00 | -46.00 | 12.21 | -61.54 | -43.73 | 99 | Posterior | Right | VIIIb |
|  | 1755 | 2.97 | -8.00 | -47.00 | -49.00 | -7.23 | -48.86 | -45.64 | 377 | Posterior | Left | IX |
|  | 1755 | 2.71 | 0.00 | -59.00 | -46.00 | 0.29 | -60.64 | -45.57 | 16 | Posterior | Vermis | IX |
|  | 1755 | 3.01 | 10.00 | -58.00 | -45.00 | 8.07 | -52.21 | -45.94 | 485 | Posterior | Right | IX |
|  | 1755 | 2.63 | -13.00 | -51.00 | -40.00 | -12.42 | -52.22 | -37.88 | 24 | DN | Left | Dentate |
|  | 1755 | 3.06 | 17.00 | -56.00 | -41.00 | 17.15 | -57.05 | -35.42 | 667 | DN | Right | Dentate |
|  | 1755 | 2.82 | 11.00 | -58.00 | -26.00 | 10.76 | -57.27 | -26.00 | 4 | DN | Right | Interposed |
| Speech without gender as covariate | 1685 | 2.46 | -9.00 | -41.00 | -28.00 | -9.00 | -41.00 | -28.00 | 1 | Anterior | Left | I_IV |
|  | 1685 | 2.62 | 12.00 | -41.00 | -28.00 | 11.52 | -42.35 | -26.93 | 58 | Anterior | Right | I_IV |
|  | 1685 | 3.08 | 7.00 | -67.00 | -45.00 | 7.42 | -66.95 | -43.82 | 7 | Posterior | Vermis | VIIIa |
|  | 1685 | 3.18 | 8.00 | -66.00 | -45.00 | 8.99 | -65.90 | -44.80 | 6 | Posterior | Right | VIIIa |
|  | 1685 | 2.98 | 7.00 | -65.00 | -46.00 | 6.18 | -64.11 | -43.92 | 39 | Posterior | Vermis | VIIIb |
|  | 1685 | 3.20 | 8.00 | -64.00 | -46.00 | 12.12 | -61.50 | -43.94 | 84 | Posterior | Right | VIIIb |
|  | 1685 | 2.85 | -3.00 | -52.00 | -49.00 | -7.00 | -48.60 | -45.87 | 334 | Posterior | Left | IX |
|  | 1685 | 2.68 | 0.00 | -59.00 | -46.00 | 0.75 | -60.62 | -45.81 | 10 | Posterior | Vermis | IX |
|  | 1685 | 2.99 | 10.00 | -58.00 | -45.00 | 8.01 | -52.27 | -45.96 | 504 | Posterior | Right | IX |
|  | 1685 | 2.58 | -12.00 | -51.00 | -39.00 | -12.19 | -50.39 | -38.99 | 5 | DN | Left | Dentate |
|  | 1685 | 3.10 | 18.00 | -62.00 | -37.00 | 17.16 | -56.92 | -35.14 | 633 | DN | Right | Dentate |
|  | 1685 | 2.69 | 11.00 | -58.00 | -26.00 | 10.76 | -57.28 | -26.00 | 4 | DN | Right | Interposed |
| Speech without treatment as covariate | 1784 | 2.77 | 12.00 | -41.00 | -28.00 | 11.23 | -42.02 | -26.89 | 39 | Anterior | Right | I_IV |
|  | 1784 | 2.99 | 7.00 | -67.00 | -45.00 | 6.97 | -66.95 | -43.75 | 8 | Posterior | Vermis | VIIIa |
|  | 1784 | 3.49 | 8.00 | -66.00 | -45.00 | 8.98 | -65.51 | -45.26 | 4 | Posterior | Right | VIIIa |
|  | 1784 | 3.06 | 7.00 | -65.00 | -46.00 | 5.87 | -64.02 | -44.07 | 46 | Posterior | Vermis | VIIIb |
|  | 1784 | 3.23 | 8.00 | -65.00 | -46.00 | 12.01 | -61.54 | -44.05 | 86 | Posterior | Right | VIIIb |
|  | 1784 | 3.07 | -8.00 | -47.00 | -49.00 | -7.20 | -48.92 | -45.52 | 458 | Posterior | Left | IX |
|  | 1784 | 2.86 | 0.00 | -59.00 | -46.00 | 0.60 | -60.69 | -45.50 | 21 | Posterior | Vermis | IX |
|  | 1784 | 3.07 | 13.00 | -45.00 | -43.00 | 7.96 | -52.34 | -45.88 | 580 | Posterior | Right | IX |
|  | 1784 | 2.60 | -12.00 | -51.00 | -39.00 | -12.40 | -52.15 | -37.92 | 24 | DN | Left | Dentate |
|  | 1784 | 3.02 | 17.00 | -56.00 | -41.00 | 17.29 | -56.31 | -35.87 | 518 | DN | Right | Dentate |
| Speech without any covariate | 2604 | 2.61 | -9.00 | -41.00 | -28.00 | -9.03 | -45.34 | -25.66 | 32 | Anterior | Left | I_IV |
|  | 2604 | 2.97 | 13.00 | -41.00 | -29.00 | 11.25 | -42.57 | -26.63 | 74 | Anterior | Right | I_IV |
|  | 2604 | 2.83 | 7.00 | -67.00 | -45.00 | 6.51 | -67.02 | -42.61 | 20 | Posterior | Vermis | VIIIa |
|  | 2604 | 2.87 | 10.00 | -65.00 | -46.00 | 11.58 | -65.28 | -43.97 | 23 | Posterior | Right | VIIIa |
|  | 2604 | 3.20 | 6.00 | -66.00 | -45.00 | 6.09 | -64.01 | -43.65 | 55 | Posterior | Vermis | VIIIb |
|  | 2604 | 3.31 | 8.00 | -65.00 | -46.00 | 11.92 | -61.78 | -43.56 | 127 | Posterior | Right | VIIIb |
|  | 2604 | 3.07 | 0.00 | -49.00 | -51.00 | -6.78 | -49.08 | -45.43 | 525 | Posterior | Left | IX |
|  | 2604 | 2.80 | 0.00 | -59.00 | -46.00 | 0.97 | -60.54 | -45.46 | 21 | Posterior | Vermis | IX |
|  | 2604 | 3.18 | 10.00 | -59.00 | -45.00 | 7.98 | -52.64 | -45.41 | 771 | Posterior | Right | IX |
|  | 2604 | 2.72 | -12.00 | -51.00 | -39.00 | -11.89 | -50.79 | -37.72 | 24 | DN | Left | Dentate |
|  | 2604 | 3.09 | 17.00 | -56.00 | -41.00 | 17.38 | -57.23 | -36.15 | 931 | DN | Right | Dentate |
|  | 2604 | 2.62 | 11.00 | -58.00 | -26.00 | 11.00 | -58.00 | -26.00 | 1 | DN | Right | Interposed |

**Table 2.** VLSM results for cognition tests.

|  |  |  | **Peak MNI coordinate** | | | **Center MNI coordinate** | | |  | Localisation | | |
| --- | --- | --- | --- | --- | --- | --- | --- | --- | --- | --- | --- | --- |
|  | **Cluster size** | **T mean** | **x** | **y** | **z** | **x2** | **y2** | **z2** | **number of voxels in the cluster** | Side | lobe | Area |
| FSIQ with all covariates (age at surgery, deep nuclei presence, treatment and gender). | 2048 | 2.66 | 9.00 | -62.00 | -25.00 | 7.09 | -60.86 | -22.69 | 66 | Anterior | Right | V |
|  | 2048 | 2.76 | 3.00 | -64.00 | -29.00 | 1.92 | -64.98 | -26.32 | 201 | Posterior | Vermis | VI |
|  | 2048 | 2.67 | 14.00 | -69.00 | -32.00 | 11.28 | -66.70 | -28.43 | 32 | Posterior | Right | VI |
|  | 2048 | 2.47 | 11.00 | -71.00 | -31.00 | 10.50 | -71.50 | -31.25 | 4 | Posterior | Right | CrusI |
|  | 2048 | 3.38 | 3.00 | -72.00 | -37.00 | 2.76 | -71.57 | -35.16 | 18 | Posterior | Vermis | CrusII |
|  | 2048 | 2.60 | 4.00 | -73.00 | -39.00 | 8.76 | -72.12 | -36.55 | 14 | Posterior | Right | CrusII |
|  | 2048 | 2.79 | 4.00 | -70.00 | -35.00 | 2.60 | -67.49 | -31.17 | 84 | Posterior | Vermis | VIIb |
|  | 2048 | 2.89 | 9.00 | -69.00 | -42.00 | 9.58 | -69.07 | -37.58 | 139 | Posterior | Right | VIIb |
|  | 2048 | 3.33 | 1.00 | -71.00 | -41.00 | 2.81 | -67.20 | -37.85 | 585 | Posterior | Vermis | VIIIa |
|  | 2048 | 2.88 | 9.00 | -68.00 | -42.00 | 10.67 | -66.31 | -41.11 | 92 | Posterior | Right | VIIIa |
|  | 2048 | 3.24 | 5.00 | -66.00 | -44.00 | 2.84 | -63.62 | -41.46 | 348 | Posterior | Vermis | VIIIb |
|  | 2048 | 3.26 | 8.00 | -63.00 | -44.00 | 8.98 | -63.21 | -43.10 | 31 | Posterior | Right | VIIIb |
|  | 2048 | 2.66 | -3.00 | -56.00 | -41.00 | -2.45 | -54.96 | -41.56 | 26 | Posterior | Left | IX |
|  | 2048 | 2.76 | 2.00 | -62.00 | -45.00 | 0.05 | -58.65 | -41.51 | 145 | Posterior | Vermis | IX |
|  | 2048 | 3.02 | 7.00 | -62.00 | -43.00 | 6.24 | -60.30 | -43.28 | 57 | Posterior | Right | IX |
|  | 2048 | 2.72 | 15.00 | -67.00 | -39.00 | 13.00 | -65.87 | -33.73 | 200 | DN | Right | Dentate |
|  | 2048 | 2.62 | 10.00 | -58.00 | -25.00 | 9.02 | -58.00 | -25.82 | 6 | DN | Right | Interposed |
| FSIQ without age at surgery as covariate | 1740 | 2.66 | 5.00 | -62.00 | -23.00 | 3.91 | -61.70 | -21.91 | 23 | Anterior | Right | V |
|  | 1740 | 2.77 | 2.00 | -68.00 | -27.00 | 1.86 | -64.90 | -26.30 | 188 | Posterior | Vermis | VI |
|  | 1740 | 3.45 | 3.00 | -72.00 | -37.00 | 2.52 | -71.67 | -35.51 | 15 | Posterior | Vermis | CrusII |
|  | 1740 | 4.28 | 4.00 | -73.00 | -39.00 | 4.00 | -73.00 | -39.00 | 1 | Posterior | Right | CrusII |
|  | 1740 | 2.73 | 5.00 | -68.00 | -34.00 | 2.66 | -67.52 | -31.17 | 82 | Posterior | Vermis | VIIb |
|  | 1740 | 2.78 | 9.00 | -69.00 | -42.00 | 9.78 | -68.93 | -38.63 | 81 | Posterior | Right | VIIb |
|  | 1740 | 3.35 | 1.00 | -71.00 | -41.00 | 2.67 | -67.35 | -38.09 | 561 | Posterior | Vermis | VIIIa |
|  | 1740 | 2.84 | 8.00 | -66.00 | -45.00 | 10.59 | -66.67 | -41.62 | 54 | Posterior | Right | VIIIa |
|  | 1740 | 3.21 | 5.00 | -66.00 | -44.00 | 2.44 | -63.56 | -41.50 | 387 | Posterior | Vermis | VIIIb |
|  | 1740 | 3.34 | 8.00 | -63.00 | -44.00 | 8.72 | -63.27 | -43.29 | 28 | Posterior | Right | VIIIb |
|  | 1740 | 2.70 | -3.00 | -56.00 | -41.00 | -2.53 | -55.25 | -42.02 | 31 | Posterior | Left | IX |
|  | 1740 | 2.78 | 2.00 | -62.00 | -45.00 | 0.35 | -58.35 | -41.51 | 182 | Posterior | Vermis | IX |
|  | 1740 | 3.01 | 7.00 | -62.00 | -43.00 | 5.88 | -59.84 | -43.19 | 61 | Posterior | Right | IX |
|  | 1740 | 2.56 | 15.00 | -67.00 | -39.00 | 13.24 | -66.88 | -36.70 | 32 | DN | Right | Dentate |
|  | 1740 | 2.45 | 6.00 | -59.00 | -33.00 | 6.43 | -58.14 | -31.29 | 14 | DN | Right | Interposed |
| FSIQ without deep nuclei as covariate |  |  |  |  |  |  |  |  |  |  |  |  |
| FSIQ without gender as covariate | 1885 | 2.66 | 16.00 | -57.00 | -26.00 | 10.89 | -60.11 | -24.71 | 14 | Anterior | Right | V |
|  | 1885 | 2.70 | 2.00 | -73.00 | -27.00 | 1.87 | -67.16 | -26.49 | 169 | Posterior | Vermis | VI |
|  | 1885 | 2.72 | 12.00 | -69.00 | -29.00 | 13.47 | -65.85 | -28.50 | 92 | Posterior | Right | VI |
|  | 1885 | 2.84 | 11.00 | -72.00 | -32.00 | 10.49 | -71.52 | -31.26 | 4 | Posterior | Right | CrusI |
|  | 1885 | 3.14 | 3.00 | -72.00 | -37.00 | 2.73 | -72.52 | -32.79 | 83 | Posterior | Vermis | CrusII |
|  | 1885 | 3.12 | 4.00 | -73.00 | -39.00 | 8.35 | -72.57 | -35.54 | 17 | Posterior | Right | CrusII |
|  | 1885 | 2.76 | 4.00 | -70.00 | -35.00 | 3.18 | -67.90 | -31.32 | 74 | Posterior | Vermis | VIIb |
|  | 1885 | 3.33 | 9.00 | -69.00 | -42.00 | 9.70 | -69.24 | -37.84 | 129 | Posterior | Right | VIIb |
|  | 1885 | 3.60 | 6.00 | -68.00 | -42.00 | 3.07 | -67.79 | -38.70 | 463 | Posterior | Vermis | VIIIa |
|  | 1885 | 3.18 | 9.00 | -68.00 | -42.00 | 10.84 | -66.28 | -41.42 | 94 | Posterior | Right | VIIIa |
|  | 1885 | 3.49 | 7.00 | -64.00 | -44.00 | 3.95 | -64.05 | -42.50 | 232 | Posterior | Vermis | VIIIb |
|  | 1885 | 3.05 | 8.00 | -63.00 | -44.00 | 10.23 | -62.43 | -43.73 | 61 | Posterior | Right | VIIIb |
|  | 1885 | 2.52 | -3.00 | -53.00 | -42.00 | -2.50 | -54.00 | -40.68 | 6 | Posterior | Left | IX |
|  | 1885 | 2.81 | 2.00 | -62.00 | -45.00 | 1.51 | -59.64 | -42.83 | 58 | Posterior | Vermis | IX |
|  | 1885 | 3.06 | 6.00 | -61.00 | -42.00 | 7.30 | -60.40 | -43.91 | 87 | Posterior | Right | IX |
|  | 1885 | 2.93 | 15.00 | -67.00 | -39.00 | 14.44 | -64.93 | -33.96 | 300 | DN | Right | Dentate |
|  | 1885 | 2.75 | 10.00 | -58.00 | -25.00 | 10.45 | -58.00 | -25.45 | 2 | DN | Right | Interposed |
| FSIQ without treatment as covariate | 1743 | 2.53 | 3.00 | -64.00 | -29.00 | 1.71 | -66.68 | -27.54 | 30 | Posterior | Vermis | VI |
|  | 1743 | 3.42 | 3.00 | -72.00 | -37.00 | 2.54 | -71.67 | -35.49 | 15 | Posterior | Vermis | CrusII |
|  | 1743 | 4.25 | 4.00 | -73.00 | -39.00 | 4.00 | -73.00 | -39.00 | 1 | Posterior | Right | CrusII |
|  | 1743 | 2.62 | 4.00 | -70.00 | -35.00 | 2.22 | -67.57 | -31.38 | 64 | Posterior | Vermis | VIIb |
|  | 1743 | 2.87 | 9.00 | -69.00 | -42.00 | 9.95 | -68.92 | -38.75 | 79 | Posterior | Right | VIIb |
|  | 1743 | 3.38 | 1.00 | -71.00 | -41.00 | 2.95 | -67.31 | -38.32 | 523 | Posterior | Vermis | VIIIa |
|  | 1743 | 2.79 | 8.00 | -66.00 | -45.00 | 10.81 | -66.37 | -41.79 | 75 | Posterior | Right | VIIIa |
|  | 1743 | 3.26 | 5.00 | -66.00 | -44.00 | 2.63 | -63.47 | -41.44 | 413 | Posterior | Vermis | VIIIb |
|  | 1743 | 3.19 | 8.00 | -63.00 | -44.00 | 9.99 | -62.43 | -43.93 | 53 | Posterior | Right | VIIIb |
|  | 1743 | 2.69 | -3.00 | -56.00 | -41.00 | -2.16 | -55.57 | -43.29 | 56 | Posterior | Left | IX |
|  | 1743 | 2.83 | 2.00 | -62.00 | -45.00 | 0.26 | -58.24 | -41.62 | 226 | Posterior | Vermis | IX |
|  | 1743 | 2.88 | 7.00 | -62.00 | -43.00 | 5.81 | -59.19 | -43.83 | 166 | Posterior | Right | IX |
|  | 1743 | 2.64 | 15.00 | -67.00 | -39.00 | 13.31 | -66.87 | -37.13 | 28 | DN | Right | Dentate |
|  | 1743 | 2.50 | 6.00 | -59.00 | -33.00 | 6.43 | -58.14 | -31.29 | 14 | DN | Right | Interposed |
| FSIQ without any covariate |  |  |  |  |  |  |  |  |  |  |  |  |
| FRI with all covariates | 1618 | 2.61 | 5.00 | -62.00 | -23.00 | 3.91 | -61.70 | -21.91 | 23 | Anterior | Right | V |
|  | 1618 | 2.75 | -1.00 | -69.00 | -23.00 | 1.14 | -65.94 | -25.60 | 237 | Posterior | Vermis | VI |
|  | 1618 | 3.37 | 2.00 | -72.00 | -37.00 | 1.66 | -71.46 | -34.78 | 27 | Posterior | Vermis | CrusII |
|  | 1618 | 4.40 | 4.00 | -73.00 | -39.00 | 4.00 | -73.00 | -39.00 | 1 | Posterior | Right | CrusII |
|  | 1618 | 2.82 | 4.00 | -70.00 | -35.00 | 2.06 | -67.68 | -31.16 | 91 | Posterior | Vermis | VIIb |
|  | 1618 | 3.16 | 8.00 | -69.00 | -39.00 | 9.80 | -69.06 | -38.67 | 91 | Posterior | Right | VIIb |
|  | 1618 | 3.44 | 7.00 | -68.00 | -40.00 | 2.33 | -67.55 | -38.07 | 578 | Posterior | Vermis | VIIIa |
|  | 1618 | 3.13 | 9.00 | -68.00 | -41.00 | 10.56 | -66.37 | -40.91 | 71 | Posterior | Right | VIIIa |
|  | 1618 | 3.14 | 7.00 | -65.00 | -42.00 | 2.47 | -63.70 | -41.18 | 248 | Posterior | Vermis | VIIIb |
|  | 1618 | 3.22 | 8.00 | -63.00 | -44.00 | 8.83 | -63.17 | -42.97 | 24 | Posterior | Right | VIIIb |
|  | 1618 | 2.75 | -3.00 | -56.00 | -41.00 | -2.36 | -55.01 | -41.06 | 17 | Posterior | Left | IX |
|  | 1618 | 2.71 | -3.00 | -58.00 | -40.00 | -0.64 | -58.35 | -40.92 | 130 | Posterior | Vermis | IX |
|  | 1618 | 3.05 | 6.00 | -61.00 | -42.00 | 6.64 | -60.22 | -43.29 | 49 | Posterior | Right | IX |
|  | 1618 | 2.69 | 13.00 | -67.00 | -38.00 | 13.01 | -67.01 | -36.88 | 31 | DN | Right | Dentate |
| FRI without age at surgery as covariate | 1602 | 2.63 | 5.00 | -62.00 | -23.00 | 3.91 | -61.70 | -21.91 | 23 | Anterior | Right | V |
|  | 1602 | 2.73 | -1.00 | -67.00 | -29.00 | 1.14 | -65.91 | -25.69 | 231 | Posterior | Vermis | VI |
|  | 1602 | 3.42 | 2.00 | -72.00 | -37.00 | 1.56 | -71.57 | -34.98 | 23 | Posterior | Vermis | CrusII |
|  | 1602 | 4.44 | 4.00 | -73.00 | -39.00 | 4.00 | -73.00 | -39.00 | 1 | Posterior | Right | CrusII |
|  | 1602 | 2.73 | 4.00 | -70.00 | -35.00 | 1.98 | -67.60 | -31.15 | 92 | Posterior | Vermis | VIIb |
|  | 1602 | 3.07 | 7.00 | -71.00 | -39.00 | 9.20 | -69.04 | -38.55 | 55 | Posterior | Right | VIIb |
|  | 1602 | 3.43 | -1.00 | -71.00 | -39.00 | 2.14 | -67.59 | -38.20 | 567 | Posterior | Vermis | VIIIa |
|  | 1602 | 2.97 | 9.00 | -66.00 | -45.00 | 10.58 | -66.31 | -41.58 | 57 | Posterior | Right | VIIIa |
|  | 1602 | 3.07 | 2.00 | -65.00 | -40.00 | 1.79 | -63.65 | -41.43 | 301 | Posterior | Vermis | VIIIb |
|  | 1602 | 3.20 | 8.00 | -63.00 | -44.00 | 8.81 | -63.19 | -43.02 | 24 | Posterior | Right | VIIIb |
|  | 1602 | 2.70 | -3.00 | -56.00 | -41.00 | -2.56 | -55.50 | -41.71 | 26 | Posterior | Left | IX |
|  | 1602 | 2.75 | 2.00 | -62.00 | -45.00 | -0.38 | -58.52 | -41.36 | 155 | Posterior | Vermis | IX |
|  | 1602 | 3.06 | 6.00 | -61.00 | -42.00 | 6.51 | -60.33 | -43.37 | 46 | Posterior | Right | IX |
|  | 1602 | 2.53 | 13.00 | -67.00 | -38.00 | 13.00 | -67.00 | -38.00 | 1 | DN | Right | Dentate |
| FRI without deep nuclei as covariate |  |  |  |  |  |  |  |  |  |  |  |  |
| FRI without gender as covariate | 1589 | 3.36 | 16.00 | -57.00 | -26.00 | 16.00 | -57.00 | -26.00 | 1 | Anterior | Right | V |
|  | 1589 | 2.75 | -1.00 | -69.00 | -23.00 | 0.52 | -67.90 | -25.47 | 210 | Posterior | Vermis | VI |
|  | 1589 | 2.66 | 12.00 | -69.00 | -29.00 | 16.07 | -64.66 | -29.08 | 59 | Posterior | Right | VI |
|  | 1589 | 2.51 | 16.00 | -69.00 | -33.00 | 12.43 | -70.95 | -32.03 | 3 | Posterior | Right | CrusI |
|  | 1589 | 3.21 | 3.00 | -72.00 | -37.00 | 2.12 | -72.41 | -32.82 | 95 | Posterior | Vermis | CrusII |
|  | 1589 | 2.73 | 4.00 | -73.00 | -39.00 | 8.18 | -72.64 | -35.45 | 17 | Posterior | Right | CrusII |
|  | 1589 | 2.83 | 4.00 | -70.00 | -35.00 | 2.50 | -68.40 | -31.51 | 72 | Posterior | Vermis | VIIb |
|  | 1589 | 3.35 | 7.00 | -71.00 | -39.00 | 9.74 | -69.28 | -38.28 | 118 | Posterior | Right | VIIb |
|  | 1589 | 3.69 | 7.00 | -68.00 | -40.00 | 2.70 | -67.95 | -38.56 | 477 | Posterior | Vermis | VIIIa |
|  | 1589 | 3.30 | 9.00 | -68.00 | -41.00 | 10.84 | -66.32 | -41.14 | 85 | Posterior | Right | VIIIa |
|  | 1589 | 3.49 | 7.00 | -65.00 | -42.00 | 4.48 | -64.13 | -42.22 | 151 | Posterior | Vermis | VIIIb |
|  | 1589 | 3.27 | 8.00 | -63.00 | -44.00 | 8.97 | -63.22 | -43.26 | 30 | Posterior | Right | VIIIb |
|  | 1589 | 2.44 | 0.00 | -57.00 | -46.00 | 0.00 | -57.00 | -46.00 | 1 | Posterior | Left | IX |
|  | 1589 | 2.66 | 4.00 | -61.00 | -44.00 | 1.41 | -59.44 | -42.80 | 40 | Posterior | Vermis | IX |
|  | 1589 | 3.09 | 6.00 | -61.00 | -42.00 | 6.33 | -59.72 | -43.60 | 79 | Posterior | Right | IX |
|  | 1589 | 2.80 | 13.00 | -67.00 | -38.00 | 16.16 | -65.26 | -34.85 | 151 | DN | Right | Dentate |
| FRI without treatment as covariate | 1464 | 2.58 | 0.00 | -68.00 | -28.00 | 1.06 | -67.42 | -27.47 | 31 | Posterior | Vermis | VI |
|  | 1464 | 3.64 | 2.00 | -72.00 | -37.00 | 2.36 | -71.59 | -35.45 | 17 | Posterior | Vermis | CrusII |
|  | 1464 | 4.41 | 4.00 | -73.00 | -39.00 | 4.00 | -73.00 | -39.00 | 1 | Posterior | Right | CrusII |
|  | 1464 | 2.72 | 4.00 | -70.00 | -35.00 | 1.63 | -67.95 | -31.34 | 58 | Posterior | Vermis | VIIb |
|  | 1464 | 3.08 | 7.00 | -71.00 | -39.00 | 9.80 | -68.94 | -38.91 | 66 | Posterior | Right | VIIb |
|  | 1464 | 3.45 | 6.00 | -67.00 | -40.00 | 2.43 | -67.62 | -38.50 | 526 | Posterior | Vermis | VIIIa |
|  | 1464 | 3.05 | 9.00 | -66.00 | -45.00 | 10.59 | -66.31 | -41.52 | 61 | Posterior | Right | VIIIa |
|  | 1464 | 3.10 | 7.00 | -63.00 | -44.00 | 2.19 | -63.63 | -41.60 | 331 | Posterior | Vermis | VIIIb |
|  | 1464 | 3.47 | 8.00 | -63.00 | -44.00 | 8.81 | -63.20 | -43.10 | 26 | Posterior | Right | VIIIb |
|  | 1464 | 2.68 | -3.00 | -56.00 | -41.00 | -2.43 | -55.62 | -42.63 | 56 | Posterior | Left | IX |
|  | 1464 | 2.84 | 2.00 | -62.00 | -45.00 | -0.23 | -58.49 | -41.55 | 185 | Posterior | Vermis | IX |
|  | 1464 | 3.06 | 6.00 | -61.00 | -42.00 | 5.74 | -59.13 | -43.70 | 101 | Posterior | Right | IX |
|  | 1464 | 2.54 | 13.00 | -67.00 | -38.00 | 14.58 | -67.39 | -38.00 | 5 | DN | Right | Dentate |
| FRI without any covariate |  |  |  |  |  |  |  |  |  |  |  |  |
| WMI with all covariates |  |  |  |  |  |  |  |  |  |  |  |  |
| WMI without age at surgery as covariate |  |  |  |  |  |  |  |  |  |  |  |  |
| WMI without deep nuclei as covariate |  |  |  |  |  |  |  |  |  |  |  |  |
| WMI without gender as covariate |  |  |  |  |  |  |  |  |  |  |  |  |
| WMI without treatment as covariate |  |  |  |  |  |  |  |  |  |  |  |  |
| WMI without any covariate |  |  |  |  |  |  |  |  |  |  |  |  |
| PSI with all covariates |  |  |  |  |  |  |  |  |  |  |  |  |
| PSI without age at surgery as covariate |  |  |  |  |  |  |  |  |  |  |  |  |
| PSI without deep nuclei as covariate |  |  |  |  |  |  |  |  |  |  |  |  |
| PSI without gender as covariate |  |  |  |  |  |  |  |  |  |  |  |  |
| PSI without treatment as covariate |  |  |  |  |  |  |  |  |  |  |  |  |
| PSI without any covariate |  |  |  |  |  |  |  |  |  |  |  |  |
| VCI with all covariates |  |  |  |  |  |  |  |  |  |  |  |  |
| VCI without age at surgery as covariate |  |  |  |  |  |  |  |  |  |  |  |  |
| VCI without deep nuclei as covariate |  |  |  |  |  |  |  |  |  |  |  |  |
| VCI without gender as covariate | 1295 | 2.61 | 12.00 | -61.00 | -26.00 | 10.85 | -59.99 | -24.60 | 12 | Anterior | Right | V |
|  | 1295 | 2.61 | 2.00 | -73.00 | -27.00 | 3.05 | -68.51 | -25.99 | 88 | Posterior | Vermis | VI |
|  | 1295 | 2.66 | 10.00 | -68.00 | -29.00 | 12.31 | -66.86 | -29.08 | 42 | Posterior | Right | VI |
|  | 1295 | 2.45 | 16.00 | -69.00 | -33.00 | 12.34 | -70.99 | -32.00 | 3 | Posterior | Right | CrusI |
|  | 1295 | 2.76 | 4.00 | -71.00 | -34.00 | 3.94 | -72.65 | -32.00 | 42 | Posterior | Vermis | CrusII |
|  | 1295 | 2.53 | 4.00 | -73.00 | -39.00 | 8.47 | -72.68 | -35.49 | 16 | Posterior | Right | CrusII |
|  | 1295 | 2.60 | 5.00 | -68.00 | -34.00 | 4.20 | -68.14 | -31.21 | 22 | Posterior | Vermis | VIIb |
|  | 1295 | 2.82 | 9.00 | -69.00 | -42.00 | 10.18 | -69.26 | -38.34 | 82 | Posterior | Right | VIIb |
|  | 1295 | 3.03 | 1.00 | -71.00 | -41.00 | 3.02 | -67.79 | -39.67 | 269 | Posterior | Vermis | VIIIa |
|  | 1295 | 2.77 | 9.00 | -68.00 | -42.00 | 11.15 | -66.91 | -42.05 | 37 | Posterior | Right | VIIIa |
|  | 1295 | 3.00 | 2.00 | -63.00 | -45.00 | 1.85 | -63.98 | -42.67 | 243 | Posterior | Vermis | VIIIb |
|  | 1295 | 2.83 | 8.00 | -64.00 | -46.00 | 10.30 | -62.33 | -45.19 | 24 | Posterior | Right | VIIIb |
|  | 1295 | 2.84 | -2.00 | -55.00 | -40.00 | -2.32 | -54.71 | -40.80 | 12 | Posterior | Left | IX |
|  | 1295 | 2.77 | 2.00 | -62.00 | -46.00 | 0.15 | -58.84 | -42.03 | 136 | Posterior | Vermis | IX |
|  | 1295 | 2.75 | 7.00 | -63.00 | -45.00 | 6.10 | -60.11 | -45.13 | 64 | Posterior | Right | IX |
|  | 1295 | 2.69 | 15.00 | -67.00 | -39.00 | 14.55 | -65.99 | -33.66 | 202 | DN | Right | Dentate |
|  | 1295 | 3.01 | 10.00 | -58.00 | -25.00 | 10.00 | -58.00 | -25.00 | 1 | DN | Right | Interposed |
| VCI without treatement as covariate |  |  |  |  |  |  |  |  |  |  |  |  |
| VCI without any covariate |  |  |  |  |  |  |  |  |  |  |  |  |
